# Synthesis and biological evaluation of new pyrazolebenzene-sulphonamides as potential anticancer agents and hCA I and II inhibitors

**DOI:** 10.3906/kim-2009-37

**Published:** 2021-06-30

**Authors:** Mehtap TUĞRAK, Halise İnci GÜL, Hiroshi SAKAGAMİ, Rüya KAYA, İlhami GÜLÇİN

**Affiliations:** 1 Department of Pharmaceutical Chemistry, Faculty of Pharmacy, Atatürk University, Erzurum Turkey; 2 Division of Pharmacology, Meikai University School of Dentistry, Sakado, Saitama Japan; 3 Department of Chemistry, Faculty of Science, Atatürk University, Erzurum Turkey; 4 Central Research and Application Laboratory, Ağrı İbrahim Çeçen University, Ağrı Turkey

**Keywords:** Sulphonamide, pyrazoline, chalcone, cytotoxicity, OSCC, carbonic anhydrase

## Abstract

Cancer is a disease characterized by the continuous growth of cells without adherence to the rules that healthy normal cells obey. Carbonic anhydrase I and II (CA I and CA II) inhibitors are used for the treatment of some diseases. The available drugs in the market have limitations or side effects, which bring about the need to develop new drug candidate compound(s) to overcome the problems at issue. In this study, new pyrazole-sulphonamide hybrid compounds 4-[5-(1,3-benzodioxol-5-yl)-3-aryl-4,5-dihydro-1 *H* -pyrazol-1-yl]benzenesulphonamides (4a - 4j) were designed to discover new drug candidate compounds. The compounds 4a - 4j were synthesized and their chemical structures were confirmed using spectral techniques. The hypothesis tested was whether an introduction of methoxy and polymethoxy group(s) lead to an increased potency selectivity expression (PSE) value of the compound, which reflects cytotoxicity and selectivity of the compounds. The cytotoxicity of the compounds towards tumor cell lines were in the range of 6.7 – 400 µM. The compounds 4i (PSE_2 _= 461.5) and 4g (PSE_1 _= 193.2) had the highest PSE values in cytotoxicity assays. Ki values of the compounds were in the range of 59.8 ± 3.0 - 12.7 ± 1.7 nM towards hCA I and in the range of 24.1 ± 7.1 - 6.9 ± 1.5 nM towards hCA II. While the compounds 4b, 4f, 4g, and 4i showed promising cytotoxic effects, the compounds 4c and 4g had the inhibitory potency towards hCA I and hCA II, respectively. These compounds can be considered as lead compounds for further research.

## 1. Introduction

Cancer is a disease characterized by the continuous growth of cells without obeying the rules that normal healthy cells do. It is second amongst the reasons for death after cardiovascular diseases [1 – 4]. Based on the World Health Organization (WHO) report in 2018, 18.1 million people around the world had cancer, and 9.6 million died from the disease. It will reach 29.4 million in 2040. Although several therapeutic approaches are available, such as chemotherapy, which includes drug therapy and has great importance. The development of new chemotherapeutics is needed since the available drugs in the market have numerous side effects, resistance development to itself, or selectivity problems [1, 3, 5]. 

Oral cancer is ranked as the sixth most common malignancy worldwide [6 – 8]. The main carcinogens for oral squamous cell carcinoma (OSCC) are cigarette and alcohol products [9, 10]. Understanding the molecular mechanisms of tumorigenesis and metastasis process for OSCC can lead researchers to discover new chemotherapeutic strategies and improve the treatment of oral cancer. 

**Figure 1:**
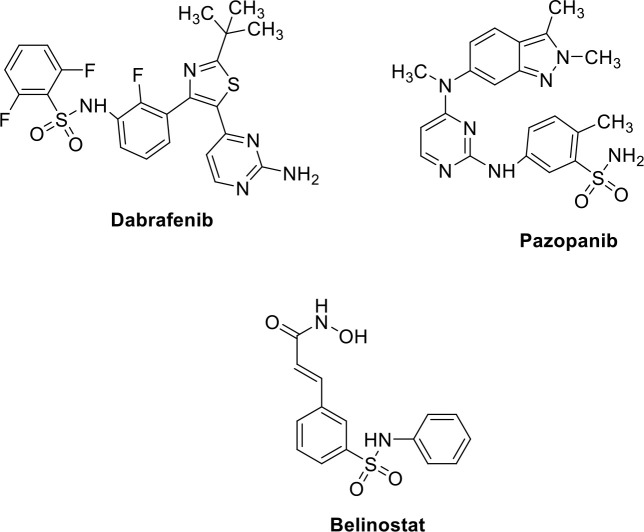
Structures of sulphonamide-bearing anticancer drugs in the clinic.

Although there are several types of anticancer therapeutic products, novel aryl sulphonamides have recently been reported to have anticancer properties and can be used to treat different types of cancers. Among them, pazopanib, a tyrosine kinase inhibitor for renal cell carcinoma and soft tissue sarcoma, belinostat, a histone deacetylase inhibitor for peripheral T-cell lymphoma, and dabrafenib, a BRAF inhibitor for metastatic melanoma, have been approved for in the clinic treatment of patients [11] (Figure 1).

As another well-known pharmacophore in drugs or bioactive compounds are pyrazoline and its analogs. Their numerous bioactivities have been reported including their anticancer/cytotoxic activities [12 – 14]. 

Our research group reported anticancer activities of many pyrazoline-based sulphonamides against OSCC lines [12 – 14]. For instance, (4-[5-(2,3,4-trimethoxyphenyl)-3-(thiophen-2-yl)-4,5-dihydro-1 *H* -pyrazol-1-yl]benzensulphonamide compound (34) showed remarkable cytotoxicity with potency-selectivity expression (PSE) and tumor specificity (TS) (10.5 and 9.5) values towards OSCC lines [15]. Other studies also supported that pyrazoline-sulphonamides hybrid compounds are good candidates to develop new anticancer drugs [13 – 15]. Besides this, we reported a large library of methoxy substituted pyrazoline derivatives since this group attracted our attention with their cytotoxic properties against OSCC lines in our previous study [16]. Many other studies reporting on the valuable anticancer properties of several mono- or poly-methoxylated chemicals towards OSCC cell lines compared to substituents other than methoxy are available [16 – 18]. 

The hybrid approach is one of the strategies to obtain a compound or a drug with increased activity in medicinal chemistry for new drug development [19]. 

Of the pharmacophores used, sulphonamide has a very well-known carbonic anhydrase (CA) inhibitory effect. CA is an enzyme that catalyzes the reversible hydration/dehydration of CO_2_/HCO_3_^− ^[20, 21] and has various roles in physiological events such as carbon dioxide and bicarbonate transport processes, respiration, pH balancing, and CO_2_ homeostasis [22, 23]. There are 16 isoforms (hCA I–XVI) that have different localizations [24, 25], of which CAs, CA I and CA II are the abundant forms. The hCA I isoform is associated with retinal and cerebral edema, and the inhibition of CA I may help cure such conditions [22, 26 – 38]. The physiologically dominant isoform is hCA II, which is another enzyme that is associated with several disease such as epilepsy, edema, glaucoma, and altitude sickness [22, 26 – 37]. Furthermore, it has also emerged in the past few years that these enzymes can be used as potential targets for designing antiinfective drugs with a novel mechanism of action [39 – 41]. Of α-class carbonic anhydrases, CA IX and CA XII are the ones that are related to tumors. In cancer cases, CA IX levels especially increase.

**Figure 2:**
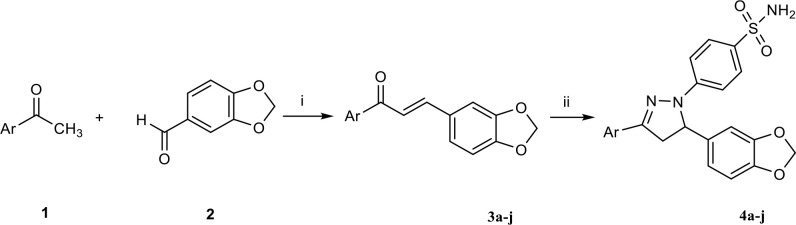
Synthesis of target pyrazoline derivatives 4a - 4j. Reagents and conditions. i: NaOH (10%), C_2_H_5_OH, rt, 24h, ii: 4-hydrazinobenzenesulphonamide hydrochloride, C2H5OH, glacial acetic acid Ar: phenyl (3a, 4a), 4-methoxyphenyl (3b, 4b), 3-methoxyphenyl (3c, 4c), 2-methoxyphenyl (3d, 4d), 3,4-dimethoxyphenyl (3e, 4e), 2,5-dimethoxyphenyl (3f, 4f), 2,4-dimethoxyphenyl (3g, 4g), 2,4,5-trimethoxyphenyl (3h, 4h), 3,4,5-trimethoxyphenyl (3i, 4i), 4-hydroxy-3-methoxyphenyl (3j, 4j).

In this study, the first aim was to synthesize pyrazolebenzene-sulphonamide hybrid compounds bearing mono- or polymethoxy (di/tri) group(s). The chemical structure of the compounds is 4-[5-(1,3-benzodioxol-5-yl)-3-aryl-4,5-dihydro-1 *H* -pyrazol-1-yl]benzenesulphonamide (4a - j, Figure 2). Secondly, the compounds (4a - j) were tested on oral squamous cell carcinoma (OSCC) and normal oral cells to find new anticancer drug candidate compounds. As a final step, it was planned to investigate the CA inhibitory effect of the compounds on hCA I and hCA II. Since CA I and II are the widely available forms of CAs, we had the opportunity facility to study them as sulphonamides are very well-known inhibitors of CAs. If impressive results are obtained is on hCA I/ II, inhibition tests towards cancer-related CA IX and CA XII isoenzymes can be considered in future studies.

## 2. Materials and methods

### 2.1. Chemistry

The chemical structures of the final compounds 4a – j were confirmed by nuclear magnetic resonance (NMR) spectra; ^1^H NMR (400MHz), ^13^C NMR (100 MHz) (Varian Mercury Plus spectrometer, Varian Inc., Palo Alto, CA, U.S.) and mass spectra (HRMS) (Shimadzu Corporation, Kyoto, Japan). Chemical shifts ( **δ** ) are reported in ppm and coupling constants ( *J* ) are expressed in hertz (Hz). Mass spectra (HMRS) for the compounds were taken using a liquid chromatography ion trap-time of flight tandem mass spectrometer (Shimadzu Corporation) equipped with an electrospray ionization (ESI) source, operating in both positive and negative ionization modes. Shimadzu’s LCMS Solution software was used for data analysis. Melting points were determined using an Electrothermal 9100/IA9100 instrument (Bibby Scientific Limited, Staffordshire, UK), which is uncorrected. Reactions were monitored by thin layer chromatography (TLC) using silica gel 60 HF254 (Merck KGaA). A solvent mixture of chloroform: methanol (4.8: 0.2) was used as a thin-layer chromatography (TLC) solvent system. DMSO- *d*_6 _(Merck) was used as a NMR solvents.

**Synthesis of chalcone compounds 3a - j, 3-(Benzo[** d **][1,3]dioxol-5-yl)-1-arylprop-2-en-1-one (Figure 2)** The title compounds were synthesized by Claisen – Schmidt condensation following to the procedure reported [25, 42] in the literature. All of the intermediate compounds (3a - j) were recorded in the literature [43 – 49]. Briefly, a mixture of suitable acetophenone (1 mmol) and benzo[ *d* ][1,3]dioxole-5-carbaldehyde (1 mmol) was dissolved in ethanol (5 mL). An aqueous sodium hydroxide solution (30%, 10 mL) was added into to the mixture under cold conditions (0 – 5 °C). After stirring overnight at room temperature, the reaction mixture was poured into an ice-water mixture and acidified with an HCl solution (10%) to pH = 6 – 7 (Figure 2). The compounds were used as a starting materials without further purification for the synthesis of pyrazoline derivatives.

#### 2.1.1. General procedure for the synthesis of pyrazolines (Figure 2, 4a - j)

A suitable chalcone (1.00 mmol) and 4-hydrazinobenzenesulphonamide hydrochloride (1.10 mmol) were dissolved in ethanol (50 mL) and then a catalytic amount of glacial acetic acid was added. The mixture was refluxed for 18 – 24 h [12, 50, 51]. Reactions were followed by thin layer chromatography (TLC). After the reaction was stopped, some of the solvent was removed under a vacuum. The obtained solid was filtered, dried at room temperature, and crystallized from methanol-diisopropylether. The compounds, 4-[5-(1,3-benzodioxol-5-yl)-3-phenyl-4,5-dihydro-1 *H* -pyrazol-1-yl]benzenesulphonamide (4a), 4-[5-(1,3-benzodioxol-5-yl)-3-(4-methoxyphenyl)-4,5-dihydro-1 *H* -pyrazol-1-yl]benzenesulphonamide (4b) and 4-[5-(1,3-benzodioxol-5-yl)-3-(3-methoxyphenyl)-4,5-dihydro-1 *H* -pyrazol-1-yl]benzenesulphonamide (4c) were reported previously [45, 52, 53]. The compound’s chemical structures were confirmed with ^1^H NMR, ^13^C NMR, and HRMS in this study. 

**4-[5-(1,3-Benzodioxol-5-yl)-3-phenyl-4,5-dihydro-1H-pyrazol-1 yl]benzenesulphonamide (4a)**

Isolated as a dark yellow solid, (yield: 30%) : mp : 196 – 198 °C (212 – 214 ºC, [45]). ^1^H NMR (400 MHz, DMSO - *d* *_6_* ) **δ** , ppm ( *J* , Hz) : 7.66 (d, 1H, *J* = 5.0 Hz, Ar-), 7.60 (d, 2H, *J* = 8.8 Hz, Ar-), 7.33 (s, 1H, Ar-), 7.13 (d, 1H, *J* = 8.6 Hz, Ar-), 7.05 – 7.01 (m, 6H, Ar-, SO_2_NH_2_), 6.87 (d, 1H, *J* = 7.9 Hz, Ar-), 6.76 – 6.72 (m, 2H, Ar-), 5.98 (s, 2H, methylene, piperonal ring), 5.56 (dd, 1H, *J* = 5.0, 11.9 Hz, pyrazoline ring), 3.93 (dd, 1H, *J* = 11.9, 17.5 Hz, pyrazoline ring), 3.19 (dd, 1H, *J* = 5.1, 17.4 Hz, pyrazoline ring); ^13^C NMR (100 MHz, DMSO - *d* *_6_* ) **δ** , ppm : 147.8, 146.6, 145.9, 145.6, 135.1, 134.9, 133.0, 128.5, 128.3, 127.9, 127.1, 118.9, 111.9, 108.6, 105.9, 101.1, 62.1, 43.6. HRMS, found, *m / z* : 421.1096 [M+4H]^+^. C_22_H_19_N_3_O_4_S. Calculated, m / z: 425.9793. 

**4-[5-(1,3-Benzodioxol-5-yl)-3-(4-methoxyphenyl)-4,5-dihydro-1H-pyrazol-1-yl]benzenesulphonamide (4b)**

Isolated as a light yellow solid (yield: 38%) : mp : 171 – 173 °C; ^1^H NMR (400 MHz, DMSO - *d* *_6_* ) **δ** , ppm ( *J* , Hz): 7.73 (d, 2H, *J* = 8.7 Hz, Ar-), 7.59 (d, 2H, *J* = 8.7 Hz, Ar-), 7.06 (d, 2H, *J* = 8.8 Hz, Ar-), 7.01 (d, 2H, *J* = 8.7 Hz, 6.86 (d, 1H, *J* = 7.9 Hz, Ar-), 6.75 - 6.72 (m, 2H, Ar-), 5.97 (s, 2H, methylene, piperonal ring), 5.51 (dd, 1H, *J* = 4.9, 11.8 Hz, pyrazoline ring), 3.88 (dd, 1H, *J* = 11.9, 17.6 Hz, pyrazoline ring), 3.80 (s, 3H, OCH_3_), 3.15 (dd, 1H, *J* = 5.0, 17.6 Hz, pyrazoline ring); ^13^C NMR (100 MHz, DMSO - *d* *_6_* ) **δ** , ppm: 160.7, 150.2, 148.2, 147.1, 146.5, 136.1, 133.1, 128.2, 127.6, 124.8, 119.5, 114.7, 112.3, 109.1, 106.5, 101.6, 62.4, 55.8, 43.6. HRMS, found, *m / z* : 452.1260 [M+H]^+^. C_23_H_21_N_3_O_5_S. Calculated, m / z: 452.1275.

**4-[5-(1,3-Benzodioxol-5-yl)-3-(3-methoxyphenyl)-4,5-dihydro-1H-pyrazol-1-yl]benzenesulphonamide (4c)**

Isolated as a cream color solid. (yield: 46%) : mp : 184 – 186 °C (219 – 221 ºC, [45]). ^1^H NMR (400 MHz, DMSO - *d* *_6_* ) **δ** , ppm ( *J* , Hz): 7.61 (d, 2H, *J* = 8.8 Hz, Ar-), 7.36 (d, 2H, *J* = 4.9 Hz, Ar-), 7.31 (s, 1H, Ar-), 7.10 (d, 2H, *J* = 8.9 Hz, Ar-), 7.05 (s, 2H, SO_2_NH_2_), 7.00 – 6.97 (m, 1H, Ar-), 6.87 (d, 1H, *J* = 7.8 Hz, Ar-), 6.76 – 6.73 (m, 2H, Ar-), 5.97 (s, 2H, methylene, piperonal ring), 5.56 (dd, 1H, *J* = 5.0, 11.9 Hz, pyrazoline ring), 3.88 (dd, 1H, *J* = 11.9, 17.7 Hz, pyrazoline ring), 3.82 (s, 3H, OCH_3_), 3.15 (dd, 1H, *J* = 5.1, 17.7 Hz, pyrazoline ring); ^13^C NMR (100 MHz, DMSO - *d* *_6_* ) **δ** , ppm: 159.9, 150.1, 148.2, 147.1, 146.3, 135.9, 133.6, 130.3, 127.6, 119.5, 119.1, 115.7, 113.9, 112.6, 111.4, 109.1, 106.4, 101.6, 62.7, 55.7, 43.4; HRMS, found, *m / z* : 452.1262 [M+H]^+^. C_23_H_21_N_3_O_5_S. Calculated, m / z: 452.1275.

**4-[5-(1,3-Benzodioxol-5-yl)-3-(2-methoxyphenyl)-4,5-dihydro-1H-pyrazol-1-yl]benzenesulphonamide (4d)**

Isolated as a cream color solid (yield: 35%) : mp : 183 – 185 °C; ^1^H NMR (400 MHz, DMSO - *d* *_6_* ) **δ** , ppm ( *J* , Hz): 7.93 (d, 2H, *J* = 7.7 Hz, Ar-), 7.60 (d, 2H, *J* = 8.8 Hz, Ar-), 7.42 – 7.38 (m, 1H, Ar-), 7.1 – 7.01 (m, 5H, Ar-, SO_2_NH_2_), 6.86 (d, 1H, *J* = 7.8 Hz, Ar), 6.76 – 6.73 (m, 2H, Ar-), 5.97 (s, 2H, methylene, piperonal ring), 5.56 (dd, 1H, *J* = 4.8, 11.8 Hz, pyrazoline ring), 3.98 (dd, 1H, *J* = 12.0, 18.3 Hz, pyrazoline ring), 3.80 (s, 3H, OCH_3_), 3.22 (dd, 1H, *J* = 5.1, 18.2 Hz, pyrazoline ring); ^13^C NMR (100 MHz, DMSO - *d* *_6_* ) **δ** , ppm: 157.9, 149.4, 148.2, 147.0, 146.5, 136.1, 133.3, 131.3, 128.8, 127.6, 121.2, 119.4, 113.9, 112.8, 112.4, 109.1, 106.4, 101.6, 62.5, 56.1, 46.8. HRMS, found, *m / z* : 452.1267 [M+H]^+^. C_23_H_21_N_3_O_5_S. Calculated, m / z: 452.1275.

**4-[5-(1,3-Benzodioxol-5-yl)-3-(3,4-dimethoxyphenyl)-4,5-dihydro-1H-pyrazol-1-yl]benzenesulphonamide (4e)**

Isolated as a cream color solid (yield: 72%) : mp : 243 – 245 °C; ^1^H NMR (400 MHz, DMSO - *d* *_6_* ) **δ** , ppm ( *J* , Hz): 7.59 (d, 2H, *J* = 8.8 Hz, Ar-), 7.42 (s, 1H, Ar-), 7.25 (d, 1H, *J* = 8.4 Hz, Ar-), 7.08 (d, 2H, *J* = 8.8 Hz, Ar-), 7.03 (s, 2H, SO_2_NH_2_), 7.00 (d, 1H, *J* = 8.5 Hz, Ar-), 6.87 (d, 1H, *J* = 7.9 Hz, Ar-), 6.76 – 6.73 (m, 2H, Ar-), 5.97 (s, 2H, methylene, piperonal ring), 5.52 (dd, 1H, *J* = 4.8, 11.8 Hz, pyrazoline ring), 3.88 (dd, 1H, *J* = 11.9, 17.6 Hz, pyrazoline ring), 3.85 (s, 3H, OCH_3_), 3.80 (s, 3H, OCH_3_), 3.17 (dd, 1H, *J* = 5.0, 17.6 Hz, pyrazoline ring); ^13^C NMR (100 MHz, DMSO - *d* *_6_* ) **δ** , ppm: 150.1, 149.9, 148.8, 147.7, 146.6, 145.9, 135.6, 132.6, 127.1, 124.4, 119.6, 118.9, 111.8, 111, 5, 108.7, 108.6, 105.9, 101.1, 61.9, 55.53, 55.50, 43.1. HRMS, found, *m / z* : 482.1358 [M+H]^+^. C_24_H_23_N_3_O_6_S. Calculated, m / z : 482.1380.

**4-[5-(1,3-Benzodioxol-5-yl)-3-(2,5-dimethoxyphenyl)-4,5-dihydro-1H-pyrazol-1-yl]benzenesulphonamide (4f)**

Isolated as a white color solid (yield: 34%) : mp : 144 – 146 °C; ^1^H NMR (400 MHz, DMSO - *d* *_6_* ) *δ* , ppm ( *J* , Hz): 7.59 (d, 2H, *J* = 8.9 Hz, Ar-), 7.46 (s, 1H, Ar-), 7.08 (d, 2H, *J* = 8.8 Hz, Ar-), 7.03 (s, 2H, SO_2_NH_2_), 7.00 – 6.97 (m, 2H, Ar-), 6.86 (d, 1H, *J* = 7.9 Hz, Ar-), 6.76 – 6.72 (m, 2H, Ar-), 5.97 (s, 2H, methylene, piperonal ring), 5.48 (dd, 1H, *J* = 5.0, 11.9 Hz, pyrazoline ring), 3.97 (dd, 1H, *J* = 12.1, 18.4 Hz, pyrazoline ring), 3.78 (s, 3H, OCH_3_), 3.75 (s, 3H, OCH_3_), 3.22 (dd, 1H, *J* = 5.2, 18.3 Hz, pyrazoline ring); ^13^C NMR (100 MHz, DMSO - *d* *_6_* ) **δ** , ppm: 153.1, 151.9, 148.6, 147.7, 146.6, 145.9, 135.6, 132.9, 127.1, 121.3, 118.9, 116.3, 113.9, 112.7, 112.0, 108.6, 105.9, 101.1, 62.1, 56.3, 55.5, 46.1. HRMS, found, *m / z* : 482.1380 [M+H]^+^. C_24_H_23_N_3_O_6_S. Calculated, m / z: 482.1360. 

**4-[5-(1,3-Benzodioxol-5-yl)-3-(2,4-dimethoxyphenyl)-4,5-dihydro-1H-pyrazol-1-yl]benzenesulphonamide (4g)**

 Isolated as a cream color solid (yield: 52%) : mp : 134 – 136 °C; ^1^H NMR (400 MHz, DMSO - *d* *_6_* ) **δ** , ppm ( *J* , Hz): 7.86 (d, 1H, *J* = 9.2 Hz, Ar-), 7.58 (d, 2H, *J* = 8.9 Hz, Ar-), 7.08 – 6.96 (m, 4H, Ar-, SO_2_NH_2_), 6.86 (d, 1H, *J* = 7.8 Hz, Ar-), 6.75 – 6.72 (m, 2H, Ar-), 6.65 – 6.62 (m, 2H, Ar-), 5.97 (s, 2H, methylene, piperonal ring), 5.42 (dd, 1H, *J* = 4.9, 11.8 Hz, pyrazoline ring), 3.92 (dd, 1H, *J* = 12.1, 19.2 Hz, pyrazoline ring), 3.82 (s, 3H, OCH_3_), 3.80 (s, 3H, OCH_3_), 3.18 (dd, 1H, *J* = 5.1, 18.2 Hz, pyrazoline ring); ^13^C NMR (100 MHz, DMSO - *d* *_6_* ) **δ** , ppm: 162.3, 159.3, 149.4, 148.2, 147.0, 146.6, 136.3, 132.9, 129.9, 127.6, 119.4, 113.9, 112.2, 109.1, 106.7, 106.4, 101.5, 99.2, 62.3, 56.2, 55.9, 46.8. HRMS, found, *m / z* : 482.1380 [M+H]^+^. C_24_H_23_N_3_O_6_S. Calculated, m / z: 482.1359.

**4-[5-(1,3-Benzodioxol-5-yl)-3-(2,4,5-trimethoxyphenyl)-4,5-dihydro-1H-pyrazol-1-yl]benzenesulphonamide (4h)**

Isolated as a yellow color solid (yield: 49%) : mp : 191 – 193 °C; ^1^H NMR (400 MHz, DMSO - *d* *_6_* ) **δ** , ppm ( *J* , Hz): 7.57 (d, 2H, *J* = 8.9 Hz, Ar-), 7.49 (s, 1H, Ar-), 7.06 – 7.01 (m, 4H, Ar-, SO_2_NH_2_), 6.86 (d, 1H, *J* = 7.9 Hz, Ar-), 6.75 – 6.72 (m, 3H, Ar-), 5.97 (s, 2H, methylene, piperonal ring), 5.43 (dd, 1H, *J* = 4.9, 11.8 Hz, pyrazoline ring), 3.95 (dd, 1H, *J* = 11.9, 18.3 Hz, pyrazoline ring), 3.84 (s, 3H, OCH_3_), 3.80 (s, 3H, OCH_3_), 3.79 (s, 3H, OCH_3_), (one of the proton peaks of the pyrazoline ring was under DMSO solvent peak); ^13^C NMR (100 MHz, DMSO - *d* *_6_* ) **δ** , ppm: 153.3, 151.8, 149.3, 148.2, 147.0, 146.5, 143.4, 136.3, 132.9, 127.5, 119.4, 112.3, 112.2, 111.5, 109.1, 106.4, 101.6, 99.0, 62.4, 57.0, 56.7, 56.2, 46.8. HRMS, found, *m / z* : 512.1486 [M + H]^+^. C_25_H_25_N_3_O_7_S. Calculated, m / z: 512.1478.

**4-[5-(1,3-Benzodioxol-5-yl)-3-(3,4,5-trimethoxyphenyl)-4,5-dihydro-1H-pyrazol-1-yl]benzenesulphonamide (4i)**

Isolated as a cream color solid. (yield: 54%) : mp : 212 – 214 °C; ^1^H NMR (400 MHz, DMSO - *d* *_6_* ) **δ** , ppm ( *J* , Hz): 7.59 (d, 2H, *J* = 8.9 Hz, Ar-), 7.11 – 7.04 (m, 6H, Ar-, SO_2_NH_2_), 6.87 (d, 1H, *J* = 7.8 Hz, Ar-), 6.75 – 6.72 (m, 2H, Ar-), 5.98 (s, 2H, methylene, piperonal ring), 5.57 (dd, 1H, *J* = 4.7, 11.9 Hz, pyrazoline ring), 3.90 (dd, 1H, *J* = 12.0, 17.6 Hz, pyrazoline ring), 3.85 (s, 6H, OCH_3_), 3.70 (s, 3H, OCH_3_), 3.26 (dd, 1H, *J* = 4.9, 17.8 Hz, pyrazoline ring); ^13^C NMR (100 MHz, DMSO - *d* *_6_* ) **δ** , ppm: 153.0, 149.8, 147.7, 146.6, 145.8, 138.7, 135.5, 132.9, 127.3, 127.1, 118.9, 111.9, 108.6, 105.9, 103.5, 101.0, 62.1, 60.1, 55.9, 43.1. HRMS, found, *m / z* : 512.1486 [M+H]^+^. C_25_H_25_N_3_O_7_S. Calculated, m / z: 512.1480.

**4-[5-(1,3-Benzodioxol-5-yl)-3-(4-hydroxy-3-methoxyphenyl)-4,5-dihydro-1H-pyrazol-1-yl]benzenesulphonamide (4j)**

 Isolated as a dark yellow solid (yield: 54%) : mp : 161 – 163 °C; ^1^H NMR (400 MHz, DMSO - *d* *_6_* ) **δ** , ppm ( *J* , Hz): 9.56 (bs, 1H, OH), 7.58 (d, 2H, *J* = 8.8 Hz, Ar-), 7.37 (s, 1H, Ar-), 7.16 (d, 2H, *J* = 8.1 Hz, Ar-), 7.06 – 7.03 (m, 4H, Ar-, SO_2_NH_2_), 6.86 (d, 1H, *J* = 7.8 Hz, Ar-), 6.75 – 6.72 (m, 2H, Ar-), 5.98 (s, 2H, methylene, piperonal ring), 5.50 (dd, 1H, *J* = 4.7, 11.7 Hz, pyrazoline ring), 3.85 (s, 6H, OCH_3_), 3.15 (dd, 1H, *J* = 4.9, 17.6 Hz, pyrazoline ring), (one of the proton peaks of the pyrazoline ring was under DMSO solvent peak); ^13^C NMR (100 MHz, DMSO - *d* *_6_* ) **δ** , ppm: 150.7, 148.9, 148.23, 148.20, 147.0, 146.5, 136.1, 132.9, 127.6, 123.6, 120.4, 119.4, 115.9, 112.2, 110.0, 109.1, 106.4, 101.6, 62.3, 56.1, 43.7. HRMS, found, m / z: 468.1224 [M+H]^+^. C_23_H_21_N_3_O_6_S. Calculated, m /z: 468.1205.

### 2.2. Biological assays 

#### 2.2.1. Cytotoxicity assay

The cytotoxicity of the compounds 4a - 4j were assayed towards human tumor cell lines [gingival carcinoma (Ca9–22), oral squamous cell carcinoma (HSC-2)] and human normal oral cells [gingival fibroblasts (HGF), and periodontal ligament fibroblasts (HPLF)] as described [42, 54 – 56]. In brief, all cells were cultured in Dulbecco’s modified eagle’s medium (DMEM) supplemented with 10% fetal bovine serum (FBS). The following concentrations of the compounds in dimethylsulfoxide (DMSO) were added to the medium and incubated at 37 **°** C for 48 h: 4a - 4j (0.32, 1, 3.2, 10, 31.6, 100, 316 and 1000 mmol/L), melphalan (3.12, 6.25, 12.5, 25. 50, 100, 200 and 400 mmol/L) and 5-FU (7.8, 15.6, 31.3, 62.5, 125, 250, 500 and 1000 mmol/L). The media that contained the same concentration of DMSO (0.0078, 0.156, 0.03125, 0.0625, 0.125, 0.25, 0.5 or 1%) were used as controls since DMSO above 0.25% is cytotoxic. The viable cell numbers were determined by the 3-(4,5-dimethylthiazol-2-yl)-2,5-diphenyltetrazolium bromide (MTT) assay. The CC_50_ values were determined from dose-response curves. 

#### 2.2.2. Carbonic anhydrase enzyme assay

Human CA isoforms (hCAI and hCAII) were purified by the sepharose - 4B – L - tyrosine sulfanilamide affinity segregation method as reported [57, 58]. The Bradford technique was used to measure protein concentrations at 595 nm [59]. Inhibitory effects of the compounds were investigated by measuring the esterase activity according to Verpoorte et al. [60] as described in previous studies [61 – 63]. The hCA activity was determined by measuring the conversion of the *p* -nitrophenyl acetate substrate to *p* -nitro phenolate at 348 nm by the spectrophotometer (UV - VIS Spectrophotometer, UV mini-1240, Shimadzu Corporation) [64]. Acetazolamide (AZA) was used as a control drug. The Lineweaver–Burk plot was used to calculate inhibition constants (K_i_) of the compounds [65] by using the following equation:

Vmax=VImax1+[I]Ki

V_max_, maximal velocity; VI_max_, maximal velocity of inhibitor; I, inhibitor; K_i_, the inhibitor constant.

Lineweaver–Burk graphics are presented as a supplementary file.

## 3. Results and discussion

### 3.1. Chemistry

The compounds (4a - j), 4-[5-(1,3-benzodioxol-5-yl)-3-aryl-4,5-dihydro-1 *H* -pyrazol-1-yl]benzenesulphonamide, were synthesized successfully. The aryl part was changed as phenyl (4a), 4-methoxyphenyl (4b), 3-methoxyphenyl (4c), 2-methoxyphenyl (4d), 3,4-dimethoxyphenyl (4e), 2,5-dimethoxyphenyl (4f), 2,4-dimethoxyphenyl (4g), 2,4,5-trimethoxyphenyl (4h), 3,4,5-trimethoxyphenyl (4i), 3-methoxy-4-hydroxyphenyl (4j). The chemical structure of the final pyrazoline compounds were elucidated by ^1^H NMR, ^13^C NMR, and HRMS. 

The NMR data proved that the target pyrazoline ring was successfully synthesized under the reaction conditions applied. A peak belonging to the proton on the fifth position of the pyrazoline ring was seen in the range of 5.57 – 5.42 ppm as double doublet (dd). Also, one of the protons on the fourth position of pyrazoline was seen in the range of 3.98 – 3.88 ppm while another proton was observed in the range of 3.22 – 3.15 ppm as dd. Two aliphatic carbons of pyrazoline (C-4 and C-5) also appeared in the range of 62.4 – 43.1 ppm in ^13^C NMR spectra as expected. HRMS results were found compatible with predicted and calculated values for the compounds.

### 3.2. Cytotoxicity

Cytotoxicity’s of the compounds 4a - 4j were evaluated towards Ca9–22, HSC-2, HSC-3, and HSC-4 human oral squamous cell carcinoma cell lines as tumor cell lines and HGF, HPLF, and HPC human normal oral cells as nontumor cells (Table 1) according to the procedure in the literature [42, 54 – 56]. 5-Fluorouracil (5-FU) was used as a reference anticancer drug. 

**Table 1:** Cytotoxicity results of pyrazoline derivatives (4a - 4j).

Tumor cell lines CC50 (μM) Non-tumor cell lines CC50 (μM)																			
	Ca9-22	SI	HSC-2	SI	HSC-3	SI	HSC-4	SI	mean	SD	HGF	HPLF	HPC	mean	SD	TS1 TS2		PSE1	PSE2
	A								B		C			D		D/B	C/A	(D/B2)×100	(C/A2)x100
4a	17.1	3.1	26.1	2.0	20.8	2.5	26.1	2	22.5	4.4	56.3	37.6	63.2	52.4	13.3	2.3	3.3	10.3	19.3
4b	14.0	21.4	18.0	16.7	16.2	18.5	18.2	16.5	16.6	2.0	400	400	98.7	299.6	174.0	18	28.6	108.6	205.1
4c	19.5	6.1	18.3	6.5	13.9	8.6	18.0	6.6	17.4	2.4	38.2	178	140	118.7	72.3	6.8	2.0	39.1	10.1
4d	13.8	2.7	17.1	2.2	12.4	3.0	14.9	2.5	14.6	2.0	36.9	39.7	37	37.8	1.6	2.6	2.7	17.9	19.5
4e	47.2	8.5	400	1.0	61.0	6.6	400	1.0	227	199.8	400	400	400	400	0.0	1.8	8.5	0.8	17.9
4f	9.7	28.2	15.7	17.4	11.5	23.8	12.0	22.8	12.2	2.5	400	276.3	147	274.4	126.5	22.4	41.2	183.6	425.1
4g	9.9	25.8	13.5	18.9	9.8	26.0	12.7	20.1	11.5	1.9	400	234.3	131	255.1	135.7	22.2	40.3	193.2	405.4
4h	400	1.0	400	1.0	21.3	18.8	21.8	18.3	211	218.5	400	400	400	400	0.0	1.9	1.0	0.9	0.3
4i	9.3	30.6	30.9	9.2	11.3	25.2	6.7	42.5	14.6	11.0	400	83.7	371.3	285	174.9	19.6	43	134.6	461.5
4j	17	3.8	26.7	2.4	26.3	2.5	16.8	3.9	21.7	5.6	76.7	64.7	54.3	65.2	11.2	3.0	4.5	13.8	26.4
5-FU	22	44.8	261	3.8	7.8	126.4	12.5	78.9	75.8	123.6	1000	1000	958.3	986.1	24.1	13.0	45.4	17.1	206

First, the question that whether the compounds have cytotoxic/anticancer properties should be answered. The cytotoxicities of the compounds towards tumor cell lines were in the range of 6.7 – 400 µM (Table 1). This shows that the compounds had anticancer properties. The compounds having more potent cytotoxicity than 5-FU and their times of potency (shown in the parenthesis) were as follows towards cell lines: Ca9–22 cell line: 4a (1.3), 4b (1.6), 4d (1.6), 4f (2.3), 4g (2.2), 4i (2.4), and 4j (1.3), and HSC-2 cell line: 4a (10), 4b (14.5), 4c (14.3), 4d (15.3), 4f (16.6), 4g (19.3), 4i (8.4), 4j (9.8). On the other hand, compounds did not show considerable cytotoxicity towards HSC-3 and HSC-4 cell lines, except compound 4i towards HSC-4.

As previously mentioned in the introduction, novel anticancer drugs that show less side effects and higher selectivity towards cancer cells urgently need to be developed [1, 3, 5]. Normal cells surround tumor cells in humans. Consequently, candidate compounds that are planned to be used in future clinical applications should show higher cytotoxicity against tumor cells rather than normal cells. The selectivity index (SI) value reflects this property. The SI values of the compounds were calculated towards a specific cell line as described before [55] and presented in Table 1. The SI figure being higher than 1 reflects the selectivity of the tested compound toward tumor cells, rather than a normal cell. In terms of SI figures, all compounds presented SI values of 2.6 – 30.6 towards Ca9–22 cell line (except 4h) while all compounds showed SI values of 2.0 – 18.9 towards HSC-2 cell line (except 4e, and 4h). On the other hand, all compounds showed SI values of 2.5 – 26.0 towards HSC-3 cell line while all compounds had SI values of 2.0 – 42.5 toward HSC-4 cell line (except 4e).

The tumor selectivity (TS_1_ and TS_2_) of each compound was calculated as described in previously reported literature procedures [55] and these figures are presented in Table 1. Based on the TS_1_ values, 4f and 4g which have 2,5 and 2,4-dimethoxy substituents had the highest TS_1_ values in a series with 22.4 and 22.2, respectively. On the other hand, the 3,4,5-trimethoxy substituted compound 4i (TS_1_: 19.6) had the second highest TS_1_ value. Among mono-methoxy compounds, the para-methoxy compound 4b had a TS_1_ value of 18 and was the third highest. As expected, they were in accordance with the literature findings [16 – 18]. The second calculation (TS_2_) considered the differences of sensitivity between the malignant (Ca9–22) and non-malignant (HGF) cells derived from the same tissue (gingiva). According to TS_2_ values obtained, 4i (3,4,5-trimethoxy) had the highest TS_2_ value of 43. This result was followed by 4f (2,5-dimethoxy), 4g (2,4-dimethoxy), and 4b (4-methoxy). 

As seen in both calculations poly-methoxylated compounds had higher TS value than mono derivatives’. The selectivity order changed as tri ˃ di ˃ mono or di ˃ tri ˃ mono. These findings also supported in our previous reports [16 – 18], thus the methoxylated compounds can be considered for new anticancer drug designs.

The desired properties for a lead compound are being both markedly cytotoxic and selectively toxic for tumors. To identify lead compounds of the study PSE (PSE_1_ and PSE_2_) values were calculated as shown in Table 1 [55]. PSE1 reflects general cytotoxicity and selectivity potency of the compound towards all cells used, whereas PSE_2_ seems more specified since it was considered for the same origin cells. When the compounds tested towards OSCC lines were considered in terms of PSE_2_ values of the compounds. The best poly-methoxylated compounds were 4i (with 3,4,5-trimethoxy, PSE_2 _= 461.5) ˃ 4f (with 2,5-dimethoxy, PSE_2 _= 425.1) ˃ 4g (with 2,4-dimethoxy, PSE_2 _= 405.4) while the best mono-methoxylated derivative was 4b (with 4-methoxy, PSE_2 _= 205). On the other hand, the non-substituted or non-metoxylated derivative 4a had a PSE_2_ value of 19.3. The reference anticancer drug 5-FU had a PSE_2 _value of 206, which is a similar value to 4-methoxy derivative 4b’s.

PSE **_2_** values of 4i, 4f, 4g were 2.2 (4i), 2.1 (4f) and 2.0 (4g) times more potent than the reference drug 5-FU. Mono- or poly-methoxylation increased the PSE_2_ values in 4b (4-methoxy), 4f (2,5-dimethoxy), 4g (2,4-dimethoxy), 4i (3,4,5-trimethoxy) derivatives for 10.6, 22.0, 21.0 and 24.0 times, respectively, compared to the methoxylated compound 4a. Methoxylation did not change the PSE_2_ value very much in 4d (2-methoxy), 4e (3,4-dimethoxy), 4j (4-hydroxy-3-methoxy) derivatives compared to the methylated compound 4a. Interestingly decreased PSE_2_ values were observed in 4c (3-methoxy, at half the value) and 4h (2,4,5-trimethoxy, with a dramatic decrease which is 64.3 times.

Therefore, it can be said that except for 4h and 4c, mono- or poly-methoxylation mostly increased or did not change PSE_2_ values of the compounds compared to non- methoxylated 4a. Even if small increases or decreases occurred with the nine methoxylated compounds, only three of them (33.3%) decreased PSE_2_ value, while 6 of them (66.6%) increased the PSE_2_ value. This suggests that methoxylation can be considered to be a useful modification to increase cytotoxicity and selectivity of compounds (PSE_2_) in general. Among mono-methoxylated ones, the (4b, 4c, 4d), 4-methoxy derivative (4b) had the best PSE value, of the dimethoxylated; 2,5-dimethoxylated (4f) and 2,4-dimethoxylated (4g) had the highest and best PSE values. When PSE_2_ value was considered, 2-methoxy (4d) compound had a similar PSE value to non-methoxylated 4a. Adding a methoxy group to 4-position in addition to 2-position in 4g compound caused 20.8 fold increase in cytotoxicity and selectivity (PSE_2_) compared to 4d (2-methoxy) and 2.0 fold increase compared to 4b (4-methoxy) derivatives. This means that the synergic effect was obtained in compound 4g when compared to 4d and 4b. Similarly, in compound 4f which is a 2,5-dimethoxy derivative, PSE value increased by 21.8 times compared to 4d (2-methoxy) and 42.1 times compared to 4c (3-methoxy) derivatives. The data confirmed that, this is a synergic effect of polymethoxy derivatives when compared to its corresponding mono analogs. 

The introduction of the third methoxy group into the structure in compound 4i (3,4,5-trimethoxy) increased the PSE_2_ value, which reflects cytotoxicity and selectivity. Increases in PSE_2_ were 2.3, 45.7, and 23.7 times in 4i compared to mono-methoxylated compounds 4b, 4c, and 4d. Increases were 1.1 and 1.12 times in 4i compared to dimethoxylated compounds 4f and 4g, respectively.

In addition, an introduction of an electron-donating hydroxy group which is a hydrogen bonding donor group to para positions of phenyl with a meta methoxy substituent in compound 4j, increased the PSE_2_ value 2.6 times in 4j comparing to 4c (3-methoxy). This was also found to be a useful modification for the increase in PSE_2_ value. 

When the order of PSE_1_ values was considered, it was as follows 4g (2,4-dimeyhoxy) ˃ 4f (2,5-dimeyhoxy) ˃ 4i (3,4,5-trimethoxy) ˃ 4b (4-methoxy), while it was 4i **˃** 4f **˃** 4g **˃** 4b in PSE_2_ calculation. Mono- or poly-methoxylation (di or tri) increased the PSE_2_ value of the compounds by 22.2%, while it increased the PSE_1_ value by 77.7%.

In a previous study, 4-(5-(3,4-dimethoxyphenyl)-3-(4-methoxyphenyl)-4,5-dihydro-1 *H* -pyrazol-1-yl)benzenesulphonamide compounds’ having free methoxy groups on its chemical structure were reported as cytotoxic against OSCC [13]. The compounds’ CC_50_ values were in the range of 22 – 200 **μM** , while their TS values were 0.7 and 3.4. When we compared our compound 4b that has piperonal moiety, which is a cyclic form of 3,4-dimethoxy groups in the previous compound [13], it can be expressed here using a piperonal structure, therefore making it a useful modification since it increased the tumor specificity of compound 4b (TS: 18 and 28.6) by 8 – 25 times towards OSCC. These significant outcomes indicate that a piperonal moiety may be used as a favorable group for designing new bioactive compounds in future studies.

The methoxy group is an electron-donating group and can form hydrogen bonds with enzymes. This is an important in the bioactivity of many compounds and drugs. Increases in the bioactivity of the compounds may be attributed to; proper interaction of the compound with the active site of the enzyme, the stability of this complex formed, and the adequate concentration of the biomacromolecules of the compound at the active site of the compound, which depends on the pharmacokinetic properties of that compound. The other factor affecting the cytotoxic potency can be the type of cell line used and the mechanism of action of the compound for the activity at issue. Furthermore, decreases in cytotoxicity may be attributed to the low stability of the compounds, which affect the concentration of the compound at the active side, or improper position of the compound at the active side which limits proper interaction. Unchanged bioactivity can bring the mind ineffectiveness of some groups to realize the activity in question partition coefficient of the compounds can also direct compound travel and its effect. Additionally, the size of molecules can be considered as an affecting factor since the behavior of the molecule in cells can be affected differently.

### 3.3. Carbonic anhydrase I and II inhibition

hCA I and hCA II inhibition results of the compounds are presented in Table 2 as IC_50_ (µM) and K_i_ (µM). When CA inhibitory profiles of the compounds were investigated based on the IC_50_ values, the compounds were effective at 6.6 - 30.1 µM toward hCA I while they were effective at 9.2 - 20.0 µM toward hCA II isoenzyme. The 3-methoxy-bearing compound 4c was the most effective inhibitor on hCA I and hCA II while phenyl-bearing compound 4a had the least effective toward hCA I in terms of IC_50_ values. The reference compound, acetazolamide (AZA), had IC_50_ values of 16.6 µM and 8.4 µM towards hCA I and II, respectively. The compounds 4b (1.5), 4c (2.5), 4d (1.3), 4e (1.6), 4f (1.2), 4g (2.5), 4h (1.2) times were more potent than AZA towards hCA I while all compounds had less inhibition potential than AZA towards hCA II in terms of IC_50_.

**Table 2:** The hCA I and hCA II inhibition values of the compounds (4a - 4j).

Compounds	IC50 (µM)				Ki (µM)	
	hCA I	r2	hCA II	r2	hCA I	hCA II
4a	30.1	0.9863	10.7	0.9846	59.8 ± 3.0	8.9 ± 2.3
4b	11.3	0.9881	16.3	0.9733	31.9 ± 1.9	11.5 ± 4.6
4c	6.6	0.9483	9.2	0.9595	21.5 ± 1.5	6.9 ± 1.5
4d	12.8	0.9487	10.7	0.9509	28.5 ± 1.6	8.9 ± 1.9
4e	10.2	0.9576	15.2	0.9679	25.5 ± 1.6	9.3 ± 0.6
4f	14.0	0.9607	16.3	0.9732	27.8 ± 10.1	12.3 ± 2.8
4g	6.7	0.9734	11.3	0.9623	12.7 ± 1.7	7.8 ± 2.2
4h	13.8	0.9875	14.3	0.9506	49.6 ± 1.9	9.3 ± 1.6
4i	29.7	0.9743	12.5	0.9412	52.5 ± 1.8	24.1 ± 7.1
4j	17.9	0.9459	20.0	0.9386	29.8 ± 9.4	9.3 ± 1.9
AZA*	16.58	0.9887	8.4	0.9825	30.2 ± 7.8	4.4 ± 0.6

When the inhibition constants (K_i_) were considered, K_i_ values of the compounds were in the range of 12.7 ± 1.7 µM – 59.8 ± 3.0 µM towards hCA I and in the range of 6.9 ± 1.5 µM – 24.1 ± 7.1 µM towards hCA II. The K_i_ values of AZA towards hCA I and hCA II were 30.2 ± 7.8 µM and 4.4 ± 0.6 µM, respectively. When K_i_ values were considered, the compound 4g, which has a 2,4-dimethoxy substituent, towards hCA I and compound 4c, which has a 3-methoxy substituent, towards hCA II had the best inhibition potential. Differences in inhibition potentials of the compounds may result from differences in their chemical structures and also differences in their interactions with the active site(s) of enzymes.

In another study conducted by our research group, a series of poly-methoxylated pyrazoline benzene sulphonamides were synthesized and their inhibitory effects on CAs were investigated. All compounds presented superior CA inhibitory activity compared to the reference compound, acetazolamide, on CAs with inhibition constants in the range of 30.1 – 49.2 nM against hCA I and of 23.8 – 30.1 nM against hCA II in terms of IC_50_ values, respectively [13]. Based on the literature findings, to obtain more potent hCA I, II inhibitors, and cytotoxic compounds, pyrazoline type compounds can be derived with poly-methoxylated phenyl rings such as 2,3,4-trimethoxy, 2,4,6-trimethoxy, and 2,4-dimethoxy groups. Additionally, bioisosteric heterocyclic rings such as furan and thiophen can be used instead of phenyl rings. Furthermore, molecular docking studies can be carried out to identify molecular interactions in future research.

## 4. Conclusion

A new series of pyrazole-sulphonamides, [4-[5-(1,3-benzodioxol-5-yl)-3-aryl-4,5-dihydro-1 *H* -pyrazol-1-yl]benzenesulphonamide] were synthesized and evaluated their cytotoxicities and carbonic anhydrase inhibitory potencies. The cytotoxicities of the compounds towards tumor cell lines were in the range of 6.7 – 400 µM. The compounds 4i (PSE_2 _= 461.5) and 4g (PSE_1 _= 193.2) had the highest PSE values in cytotoxicity assays. The use of methoxy substituents in different parts of the ring severely affected bioactivity. All compounds presented hCA I and hCA II inhibition potency. The compounds 4c (K_i _= 6.9 ± 1.5 µM, hCA II) and 4g (K_i_ = 12.7 ± 1.7 µM, hCA I) had the lowest K_i_ values as the best CA inhibitors. The compounds that show impressive bioactivities on the targets can be considered as lead compounds for further studies.

Supplementary Materials

## References

[ref1] (2013). Cancer-​associated bone disease. Osteoporosis International.

[ref2] (2020). Effects of doxorubicin on the heart: from molecular mechanisms to intervention strategies. European Journal of Pharmacology.

[ref3] (2020). Association of microRNA-27a rs895819 polymorphism with the risk of cancer: an updated meta-analysis. Gene.

[ref4] (2020). Therapeutic emergence of rhein as a potential anticancer drug: a review of its molecular targets and anticancer properties. Molecules.

[ref5] (2010). Estimates of worldwide burden of cancer in 2008. International Journal of Cancer.

[ref6] (2009). Global epidemiology of oral and oropharyngeal cancer. Oral Oncology.

[ref7] (2019). In-vitro cytotoxicity studies of methanolic bulb Extract of Zephyranthes Citrina on cervical cancer (Hela), breast cancer (MCF-7) and oral cancer (SCC-9). Journal of Pharmaceutical Science and Research.

[ref8] (2020). Malignant transformation of oral leukoplakia is associated with macrophage polarization. Journal of Translational Medicine.

[ref9] (1995). Betel quid chewing, cigarette smoking and alcohol consumption related to oral cancer in Taiwan. Journal of Oral Pathology and Medicine.

[ref10] (2011). betel quid and oral cancer: a prospective cohort study. Journal of Oncology.

[ref11] (2017). Synthesis, QSAR studies, and metabolic stability of novel 2-alkylthio-4-chloro-. N-(5-oxo-4.

[ref12] (2016). Synthesis and bioactivity studies on new 4-(3-(4-. -indeno[1.

[ref13] (2016). Synthesis, cytotoxicity and carbonic anhydrase inhibitory activities of new pyrazolines. Journal of Enzyme Inhibition and Medicinal Chemistry.

[ref14] (2017). Microwave-assisted synthesis and bioevaluation of new sulfonamides. Journal of Enzyme Inhibition and Medicinal Chemistry.

[ref15] (2018). New anticancer drug candidates sulfonamides as selective hCA IX or hCA XII inhibitors. Bioorganic Chemistry.

[ref16] (2019). Synthesis and bioactivities of pyrazoline benzensulfonamides as carbonic anhydrase and acetylcholinesterase inhibitors with low cytotoxicity. Bioorganic Chemistry.

[ref17] (2017). Synthesis and structure elucidation of 1-(2. Medicinal Chemistry Research.

[ref18] (2018). Cytotoxicity, apoptosis, and QSAR studies of phenothiazine derived methoxylated chalcones as anticancer drug candidates. Medicinal Chemistry Research.

[ref19] (2020). Novel analgesic agents obtained by molecular hybridization of orthosteric and allosteric ligands. Europen Journal of Pharmacology.

[ref20] (2019). Novel 2-aminopyridine liganded Pd(II) N-heterocyclic carbene complexes: synthesis, characterization, crystal structure and bioactivity properties. Bioorganic Chemistry.

[ref21] (2019). The green synthesis and molecular docking of novel N-substituted rhodanines as effective inhibitors for carbonic anhydrase and acetylcholinesterase enzymes. Bioorganic Chemistry.

[ref22] (2017). Advances in structure-based drug discovery of carbonic anhydrase inhibitors. Expert Opinion Drug Discovery.

[ref23] (2019). Synthesis and biological evaluation of bromophenol derivatives with cyclopropyl moiety: ring opening of cyclopropane with monoester. Bioorganic Chemistry.

[ref24] (2019). Synthesis of oxazolidinone from enantiomerically enriched allylic alcohols and determination of their molecular docking and biologic activities. Bioorganic Chemistry.

[ref25] (2019). Synthesis, biological evaluation and molecular docking of novel pyrazole derivatives as potent carbonic anhydrase and acetylcholinesterase inhibitors. Bioorganic Chemistry.

[ref26] (2013). Carbonic anhydrases: from biomedical applications of the inhibitors and activators to biotechnological use for CO2 capture. Journal of Enzyme Inhibition and Medicinal Chemistry.

[ref27] (2016). How many carbonic anhydrase inhibition mechanisms exist. Journal of Enzyme Inhibition and Medicinal Chemistry.

[ref28] (2012). Multiple binding modes of inhibitors to carbonic anhydrases: how to design specific drugs targeting 15 different isoforms. Chemical Reviews.

[ref29] (2004). Carbonic anhydrase inhibitors: X-ray crystallographic structure of the adduct of human isozyme II with EMATE, a dual inhibitor of carbonic anhydrases and steroid sulfatase. Bioorganic and Medicinal Chemistry Letters.

[ref30] (2005). Diuretics with carbonic anhydrase inhibitory action: a patent and literature review (. Expert Opinion Therapeutic Patent.

[ref31] (2013). Antiglaucoma carbonic anhydrase inhibitors: a patent review. Expert Opinion Therapeutic Patent.

[ref32] (2013). Antiobesity carbonic anhydrase inhibitors: a literature and patent review. Expert Opinion Therapeutic Patent.

[ref33] (2018). Carbonic anhydrases and metabolism. Metabolites.

[ref34] (2008). Anticancer carbonic anhydrase inhibitors: a patent review (. Expert Opinion Therapeutic Patent.

[ref35] (2004). Carbonic anhydrase inhibitors. Design of selective, membrane-impermeant inhibitors targeting the human tumor-associated isozyme IX. Journal of Medicinal Chemistry.

[ref36] (2016). Carbonic anhydrase inhibition and the management of neuropathic. Expert Review Neurotherapeutic.

[ref37] (2017). Design and synthesis of novel nonsteroidal anti-inflammatory drugs and carbonic anhydrase inhibitors hybrids (NSAIDs-CAIs) for the treatment of rheumatoid arthritis. Journal of Medicinal Chemistry.

[ref38] (2017). Carbonic anhydrase inhibition and the management of hypoxic tumors. Metabolites.

[ref39] (2015). Bacterial, fungal and protozoan carbonic anhydrases as drug targets. Expert Opinion Therapeutic Targets.

[ref40] (2015). An overview of the alpha-, beta- and gamma-carbonic anhydrases from bacteria: can bacterial carbonic anhydrases shed new light on evolution of bacteria?. Journal of Enzyme Inhibition and Medicinal Chemistry.

[ref41] (2018). Carbonic anhydrases from Trypanosoma cruzi and Leishmania donovani chagasi are inhibited by benzoxaboroles. Journal of Enzyme Inhibition and Medicinal Chemistry.

[ref42] (2018). New azafluorenones with cytotoxic and carbonic anhydrase inhibitory properties: 2-aryl-4-(4-hydroxyphenyl). -indeno[1.

[ref43] (2013). Synthesis, biological evaluation, 3D-QSAR studies of novel aryl-2H-pyrazole derivatives as telomerase inhibitors. Bioorganic and Medicinal Chemistry Letters.

[ref44] (2020). Discovery of new fluorescent thiazole-pyrazoline derivatives as autophagy inducers by inhibiting mTOR activity in A549 human lung cancer cells. Cell Death and Disease.

[ref45] (2019). Dihydropyrazole derivatives containing benzo oxygen heterocycle and sulfonamide moieties selectively and potently inhibit COX-2: design, synthesis, and anti-colon cancer activity evaluation. Molecules.

[ref46] (2016). QSAR, in silico docking and in vitro evaluation of chalcone derivatives as potential inhibitors for H1N1 virus neuraminidase. Medicinal Chemistry Research.

[ref47] (2019). Synthesis of some novel pyrazoline-​thiazole hybrids and their antimicrobial activities. Journal of Heterocyclic Chemistry.

[ref48] (2019). 2-​pyridones and their bioactivation in CYP1 expressing breast cancer cells. The synthesis of 4.

[ref49] (2012). Synthesis, biological evaluation, and molecular modeling of chalcone derivatives as potent inhibitors of mycobacterium tuberculosis protein tyrosine phosphatases (PtpA and PtpB). Journal of Medicinal Chemistry.

[ref50] (2016). Synthesis and carbonic anhydrase inhibitory activities of new thienyl-substituted pyrazoline benzenesulfonamides. Journal of Enzyme Inhibition and Medicinal Chemistry.

[ref51] (2017). -yl]benzenesulfonamides. Designing, synthesis and bioactivities of 4-[3-(4-hydroxyphenyl)-5-aryl-4.

[ref52] Synthesis of new pyrazoline and pyrazole derivatives. Indian Journal of Heterocyclic Chemistry.

[ref53] (2018). Methods for treating bacterial infection, U.S. Pat.

[ref54] (2019). Synthesis and biological evaluation of some new mono Mannich bases with piperazines as possible anticancer agents and carbonic anhydrase inhibitors. Bioorganic Chemistry.

[ref55] (2019). New phenolic Mannich bases with piperazines and their bioactivities. Bioorganic Chemistry.

[ref56] (2018). Synthesis and cytotoxicities of new azafluorenones with apoptotic mechanism of action and cell cycle analysis. Anti-cancer Agents and Medicinal Chemistry.

[ref57] (7R). Synthesis, characterization, anticancer, antimicrobial and carbonic anhydrase inhibition profiles of novel (3aR,4S. -4.

[ref58] (2017). Synthesis and bioactivity of several new hetaryl sulfonamides. Journal of Enzyme Inhibition Medicinal Chemistry.

[ref59] (1976). A rapid and sensitive method for the quantitation of microgram quantities of protein utilizing the principle of protein-dye binding. Analytical Biochemistry.

[ref60] (1967). Esterase activities of human carbonic anhydrases B and C. Journal of Biological Chemistry.

[ref61] (2009). Carbonic anhydrase inhibitors. Inhibition of human erythrocyte isozymes I and II with a series of antioxidant phenols. Bioorganic and Medicinal Chemistry.

[ref62] (2013). Novel sulfamides as potential carbonic anhydrase isoenzymes inhibitors. Bioorganic and Medicinal Chemistry.

[ref63] (2013). Synthesis and carbonic anhydrase inhibitory properties of sulfamides structurally related to dopamine. Bioorganic and Medicinal Chemistry.

[ref64] (2014). Oxidation of cyanobenzocycloheptatrienes: synthesis, photooxygenation reaction and carbonic anhydrase isoenzymes inhibition properties of some new benzotropone derivatives. Bioorganic and Medicinal Chemistry.

[ref65] (1934). The determination of enzyme dissociation constants. Journal of the American Chemical Society.

