# Supplementary material for: Synthesis and biological evaluation of new pyrazolebenzene-sulphonamides as potential anticancer agents and hCA I and II inhibitors
Source: Turk J Chem. 2021 Jun 30;45(3):528–39. doi: 10.3906/kim-2009-37 (PMC8326471; doi:10.3906/kim-2009-37)
Supplement: Supplementary file 1 — Supplementary Materials [file turkjchem-45-528-sup001.pdf]

KIM-2009-37_1_Suplementary_file_(Ki_Graphs_for_4a-j)_2.11.2020 (1)

hCA I için;

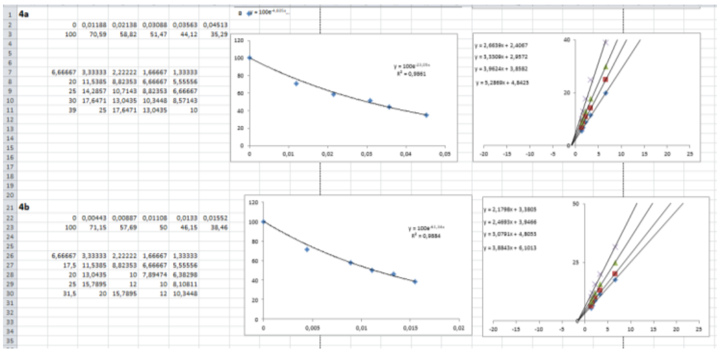

4a

0	0,01188	0,02138	0,03088	0,03563	0,04513
100	70,59	58,82	51,47	44,12	35,29
6,66667	3,33333	2,22222	1,66667	1,33333	
20	11,5385	8,82353	6,66667	5,55556	
25	14,2857	10,7143	8,82353	6,66667	
30	17,6471	13,0435	10,3448	8,57143	
39	25	17,6471	13,0435	10	

4b

0	0,00443	0,00887	0,01108	0,0133	0,01552
100	71,15	57,69	50	46,15	38,46
6,66667	3,33333	2,22222	1,66667	1,33333	
17,5	11,5385	8,82353	6,66667	5,55556	
20	13,0435	10	7,89474	6,38298	
25	15,7895	12	10	8,10811	
31,5	20	15,7895	12	10,3448	

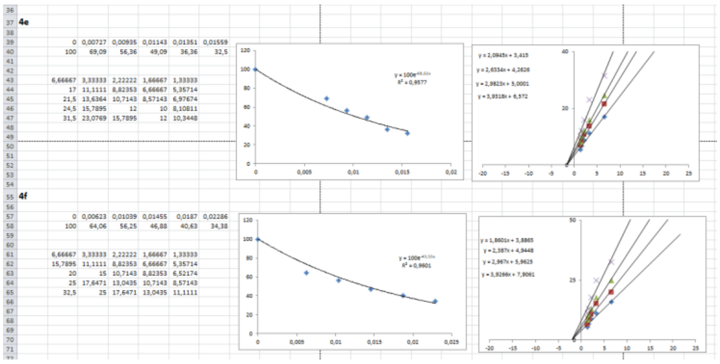

4e

0	0,00727	0,00935	0,01143	0,01351	0,01559
100	69,09	56,36	49,09	36,36	32,5
6,66667	3,33333	2,22222	1,66667	1,33333	
17	11,1111	8,82353	6,66667	5,35714	
21,5	13,6364	10,7143	8,57143	6,97674	
24,5	15,7895	12	10	8,10811	
31,5	23,0769	15,7895	12	10,3448	

4f

0	0,00623	0,01039	0,01455	0,0187	0,02286
100	64,06	56,25	46,88	40,63	34,38
6,66667	3,33333	2,22222	1,66667	1,33333	
15,7895	11,1111	8,82353	6,66667	5,35714	
20	15	10,7143	8,82353	6,52174	
25	17,6471	13,0435	10,7143	8,57143	
32,5	25	17,6471	13,0435	11,1111	

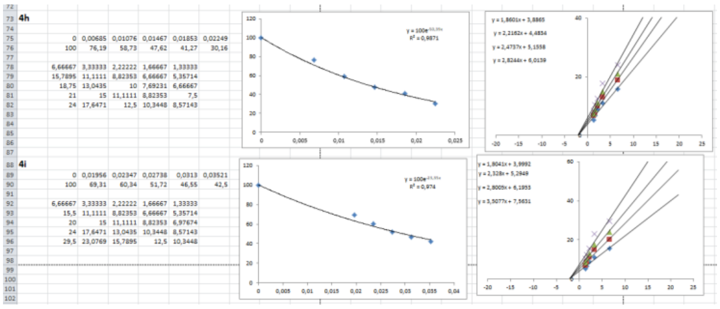

4h

0	0,00685	0,01076	0,01467	0,01853	0,02249
100	76,19	58,73	47,62	41,27	30,16
6,66667	3,33333	2,22222	1,66667	1,33333	
15,7895	11,1111	8,82353	6,66667	5,35714	
18,75	13,0435	10	7,69231	6,66667	
21	15	11,1111	8,82353	7,5	
24	17,6471	12,5	10,3448	8,57143	

4i

0	0,01956	0,02347	0,02738	0,0313	0,03521
100	69,31	60,34	51,72	46,55	42,5
6,66667	3,33333	2,22222	1,66667	1,33333	
15,5	11,1111	8,82353	6,66667	5,35714	
20	15	11,1111	8,82353	6,97674	
24	17,6471	13,0435	10,3448	8,57143	
29,5	23,0769	15,7895	12,5	10,3448	

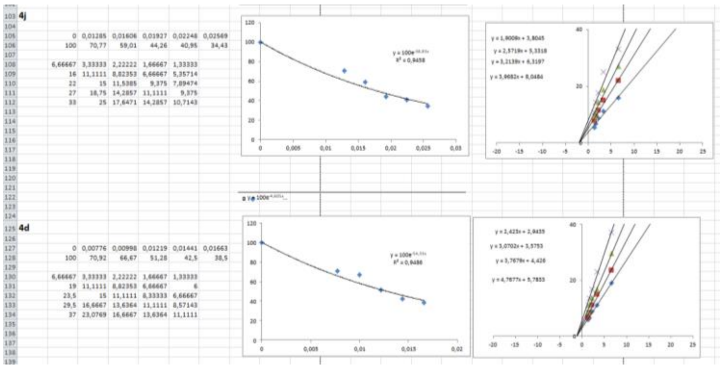

4j

0	0,01285	0,01606	0,01927	0,02248	0,02569
100	70,77	59,01	44,26	40,95	34,43
6,66667	3,33333	2,22222	1,66667	1,33333	
16	11,1111	8,82353	6,66667	5,35714	
22	15	11,5385	9,375	7,89474	
27	18,75	14,2857	11,1111	9,375	
33	25	17,6471	14,2857	10,7143	

4d

0	0,00776	0,00998	0,01219	0,01441	0,01663
100	70,92	66,67	51,28	42,5	38,5
6,66667	3,33333	2,22222	1,66667	1,33333	
19	11,1111	8,82353	6,66667	6	
23,5	15	11,1111	8,33333	6,66667	
29,5	16,6667	13,6364	11,1111	8,57143	
37	23,0769	16,6667	13,6364	11,1111	

4c

0	0,00443	0,00554	0,00665	0,00776	0,00887
100	70,77	61,54	50	42,5	35,5
6,66667	3,33333	2,22222	1,66667	1,33333	
14,8	11,1111	8,82353	6,66667	6	
16,6667	13,0435	10	7,89474	6,38298	
21,2	15	12	10,3448	8,57143	
25,8	17,6471	15	12,5	10,3448	

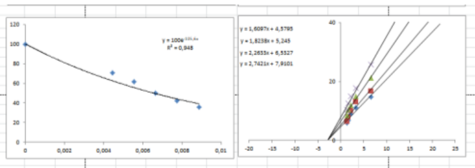

4g

0	0,00208	0,00416	0,00624	0,00831	0,01039
100	82,76	68,97	58,62	41,38	31,03
6,66667	3,33333	2,22222	1,66667	1,33333	
16	11,1111	8,82353	6,66667	6	
22	13,6364	10,7143	8,33333	10	
23,0769	16,6667	12	10,3448	8,33333	
28,2	20	14,2857	12,5	10,7143	

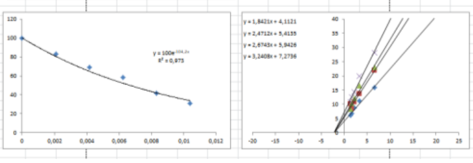

hCA II için;

4a

0	0,00475	0,0095	0,01425	0,019	0,02375
100	64,1	53,85	41,03	30,77	20,51
6,66667	3,33333	2,22222	1,66667	1,33333	
9,4	5,35714	4,6875	3,75	2,88462	
17,5	10	7,89474	6,81818	6	
25	16,6667	12,5	9,375	7,5	
35,3	21,4286	16,6667	13,6364	11,5385	

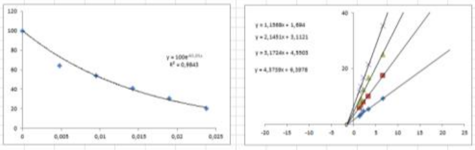

4b

0	0,01108	0,01663	0,02217	0,02771	0,03103
100	67,57	56,76	40,54	29,73	24,32
6,66667	3,33333	2,22222	1,66667	1,33333	
11,5	7,5	5,35714	4,41176	3,75	
25	13,6364	11,5385	10	7,89474	
39	30	18,75	15	10,7143	
50	37,5	25	18,75	13,6364	

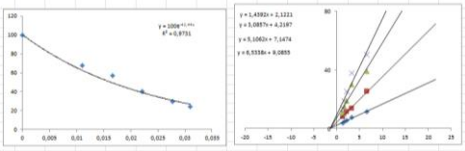

4e

0	0,00571	0,01141	0,01712	0,02282	0,02853
100	90,2	64,71	47,06	31,37	27,45
6,66667	3,33333	2,22222	1,66667	1,33333	
10,4	7,5	5,35714	4,41176	3,75	
23	15	12,5	10	7,89474	
31	21,4286	16,6667	12,5	10,7143	
38	30	21,4286	15	12,5	

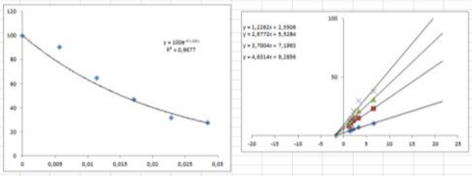

4f

0	0,00624	0,01247	0,01663	0,02078	0,02494
100	67,5	57,5	50	42,5	35
6,66667	3,33333	2,22222	1,66667	1,33333	
10,2	7,5	5,35714	4,41176	3,75	
18,75	12,5	10	8,33333	6,81818	
26	16,6667	13,6364	12,5	8,82353	
31,1	21,4286	16,6667	13,6364	11,5385	

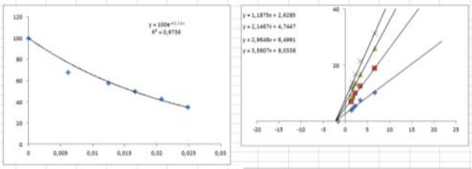

4h

0	0,00489	0,00978	0,01565	0,01956	0,02446
100	82,35	67,65	55,88	38,24	26,47
6,66667	3,33333	2,22222	1,66667	1,33333	
8	3,94737	3	2,5	2,30769	
15	8,82353	5,35714	4,6875	4,16667	
18,5	10	7,5	5,76923	5	
25	15	10	7,89474	6,52174	

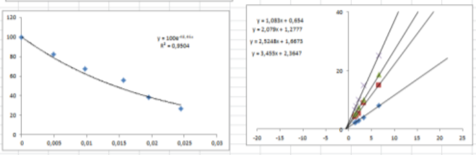

4i

0	0,00587	0,01076	0,01467	0,01859	0,0225
100	74,42	69,77	46,51	34,88	25
6,66667	3,33333	2,22222	1,66667	1,33333	
15	7,89474	6	5	4,61538	
19	12,5	8,57143	6	5	
22	15	9,67742	7,14286	5,55556	
30	20	14,2857	10	6,81818	

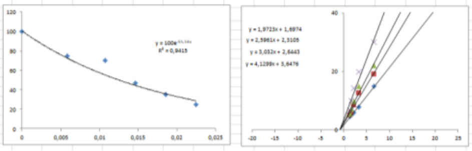

4j

0	0,0109	0,01745	0,02181	0,02617	0,03053
100	80	60,45	50,9	35,72	32,5
6,66667	3,33333	2,22222	1,66667	1,33333	
14,5	7,89474	6	5	4,61538	
44	25	18,75	15	12	
54	30	25	17,6471	13,6364	
70	50	33,3333	23,0769	15,7895	

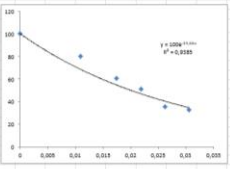

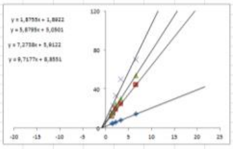

4d

0	0,00443	0,00887	0,0133	0,01773	0,02217
100	61,4	52,63	43,86	29,82	26,32
6,66667	3,33333	2,22222	1,66667	1,33333	
12,3	7,89474	6	5	4,61538	
22,8	15,7895	12	9,375	7,5	
29	18,75	15	12	10	
44	33,3333	25	17,6471	13,0435	

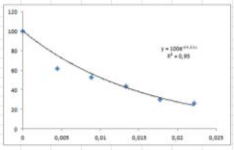

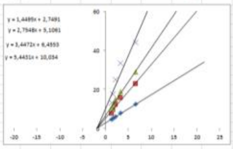

4c

0	0,00333	0,00665	0,00998	0,01219	0,01441
100	69,3	55,56	51,85	40,74	33,33
6,66667	3,33333	2,22222	1,66667	1,33333	
13,2	7,89474	6	5	4,61538	
24,5	15	11,5385	9,375	7,89474	
34	20	15,7895	13,0435	10,3448	
46,5	30	21,4286	17,6471	14,2857	

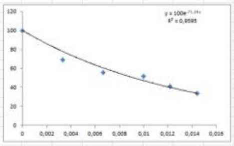

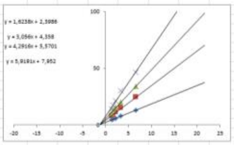

4g

0	0,00416	0,00727	0,01039	0,01351	0,01663
100	73,02	63,97	47,62	42,86	39,68
6,66667	3,33333	2,22222	1,66667	1,33333	
11,5	7,89474	6	5	4,61538	
19,5	13,6364	10,7143	8,82353	7,14286	
28	20	15,7895	12,5	10	
37,5	25	21,4286	16,6667	13,6364	

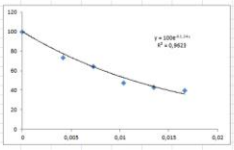

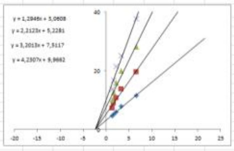

Compound 4a

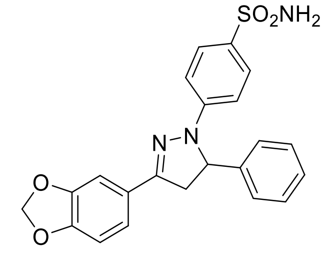

Chemical Formula: $C_{22}H_{19}N_3O_4S$
Exact Mass: 421,1096

^{1}H NMR of compound 4a

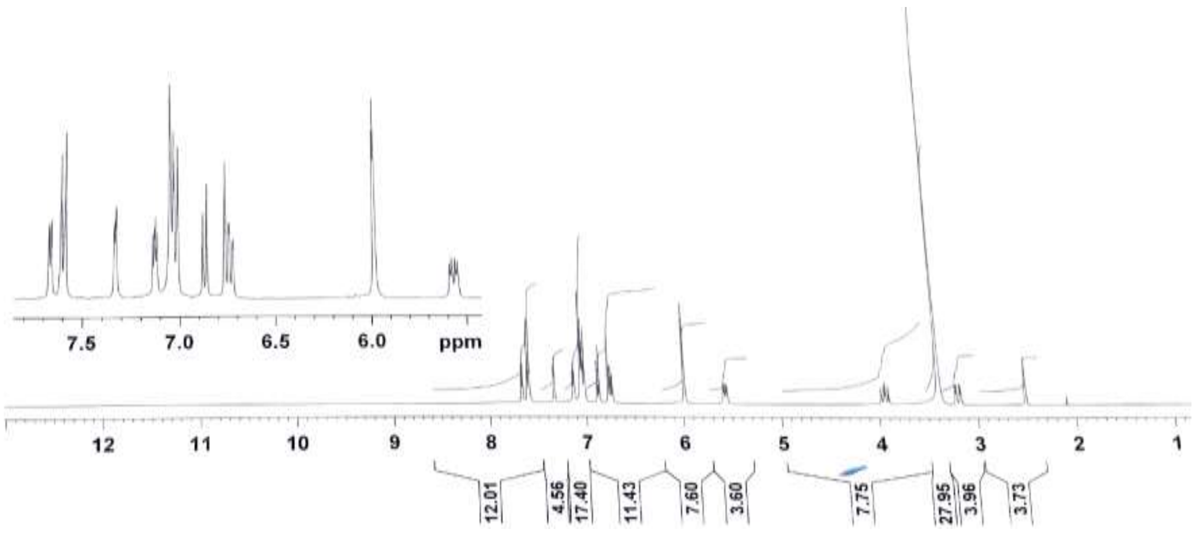

^{13} C NMR of compound 4a

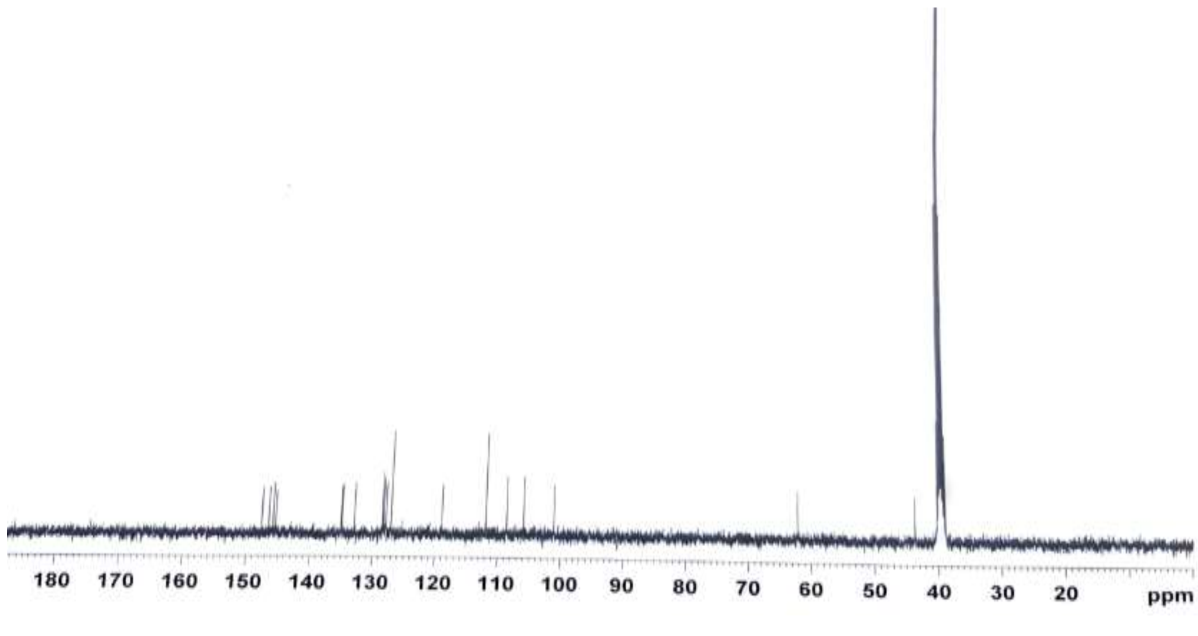

HRMS of compound 4a

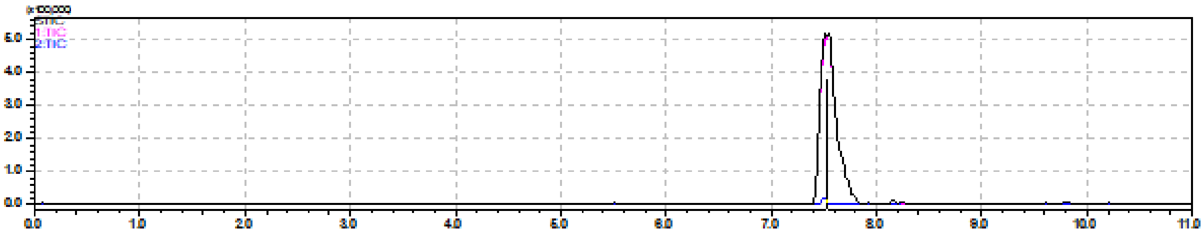

ESI (+)

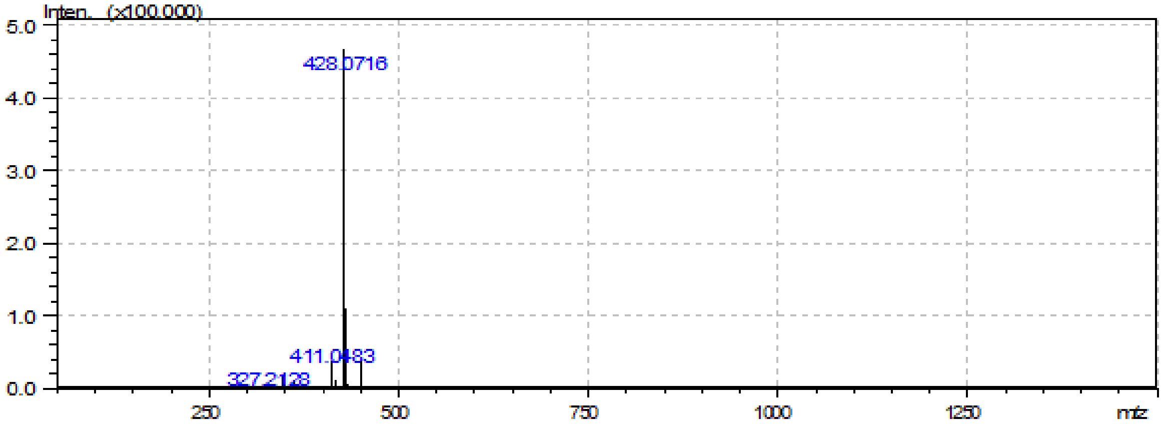

ESI (-)

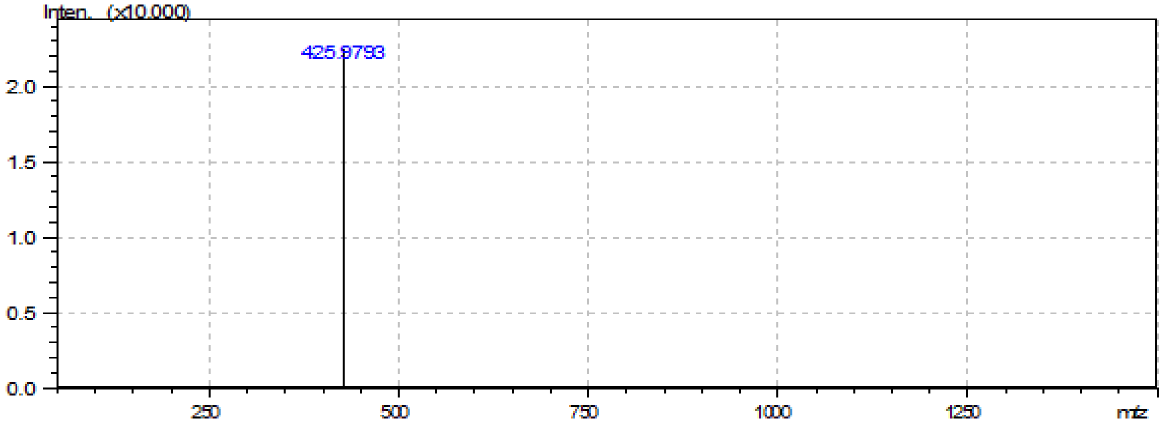

Compound 4b

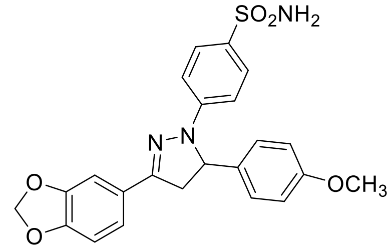

Chemical Formula: $C_{23}H_{21}N_3O_5S$
Exact Mass: 451,1202

^{1}H NMR of compound 4b

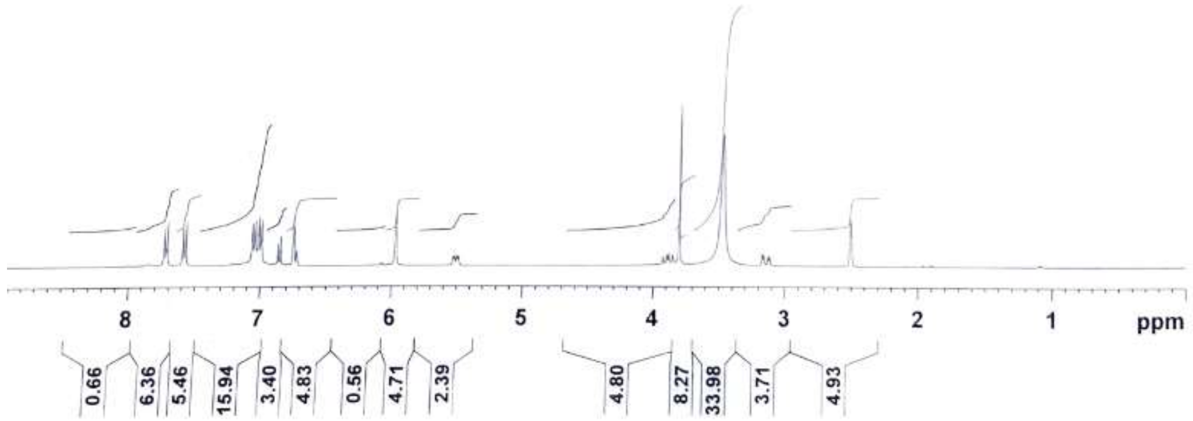

^{13} C NMR of compound 4b

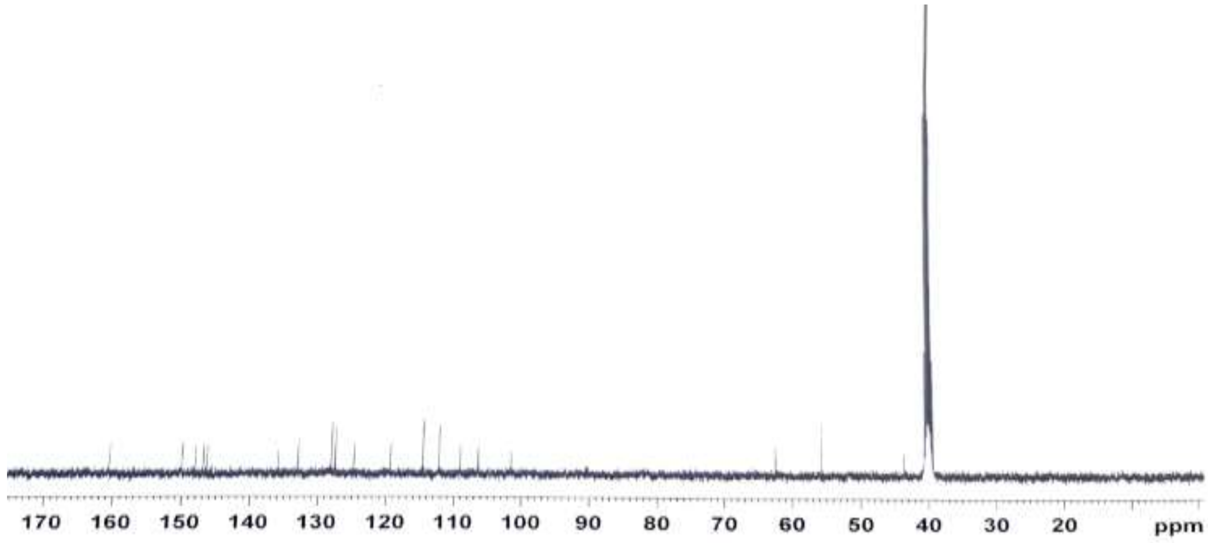

HRMS of compound 4b

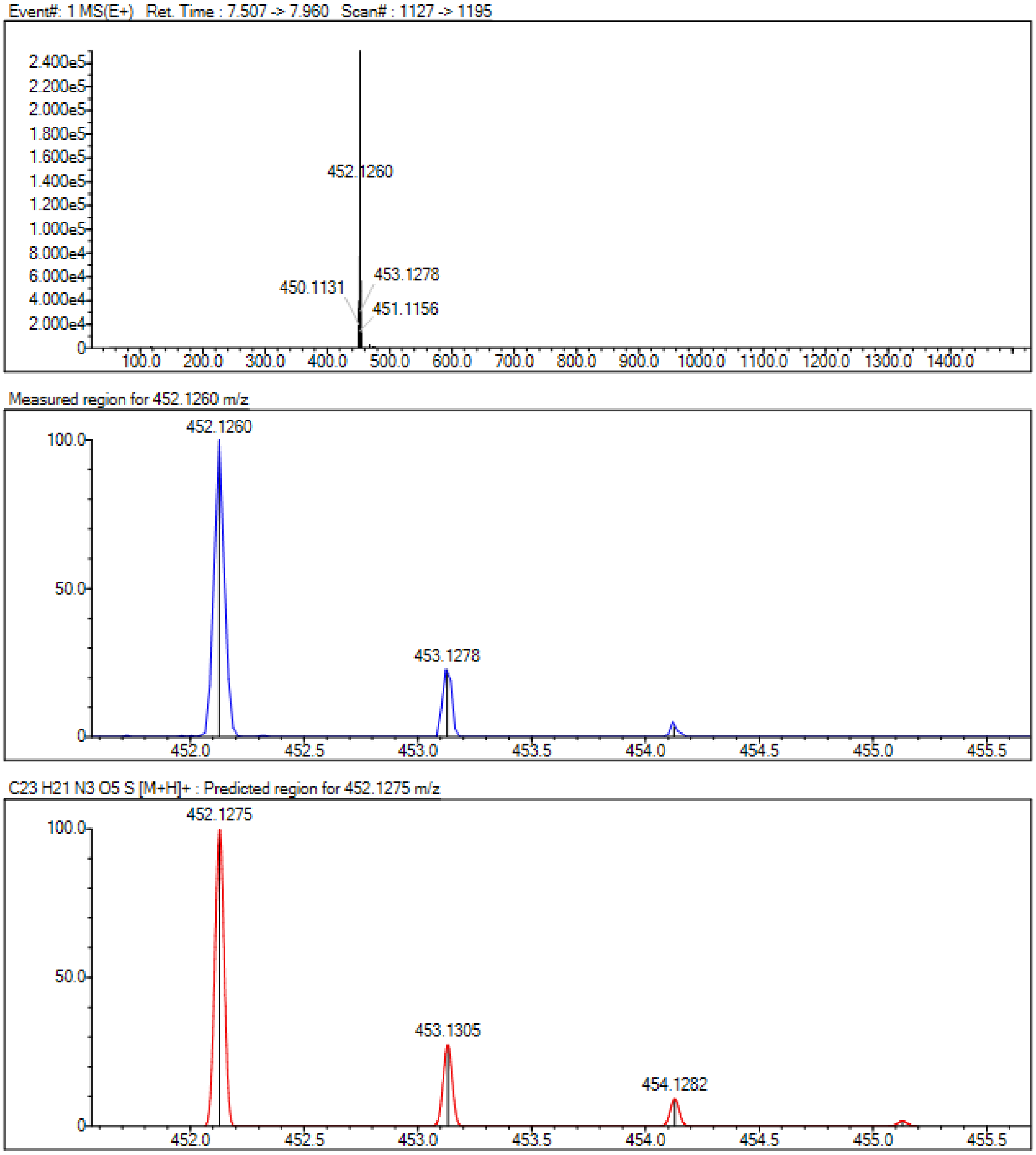

Rank	Score	Formula (M)	Ion	Meas. m/z	Pred. m/z	Df. (mDa)	Df. (ppm)	Iso	DBE
1	62.11	C23 H21 N3 O5 S	[M+H]+	452.1260	452.1275	-1.5	-3.32	65.94	15.0

Compound 4c

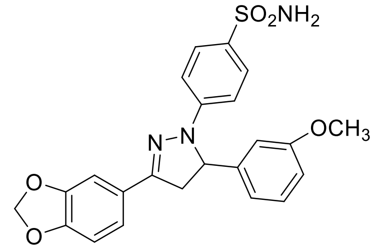

Chemical Formula: $C_{23}H_{21}N_3O_5S$
Exact Mass: 451,1202

^{1}H NMR of compound 4c

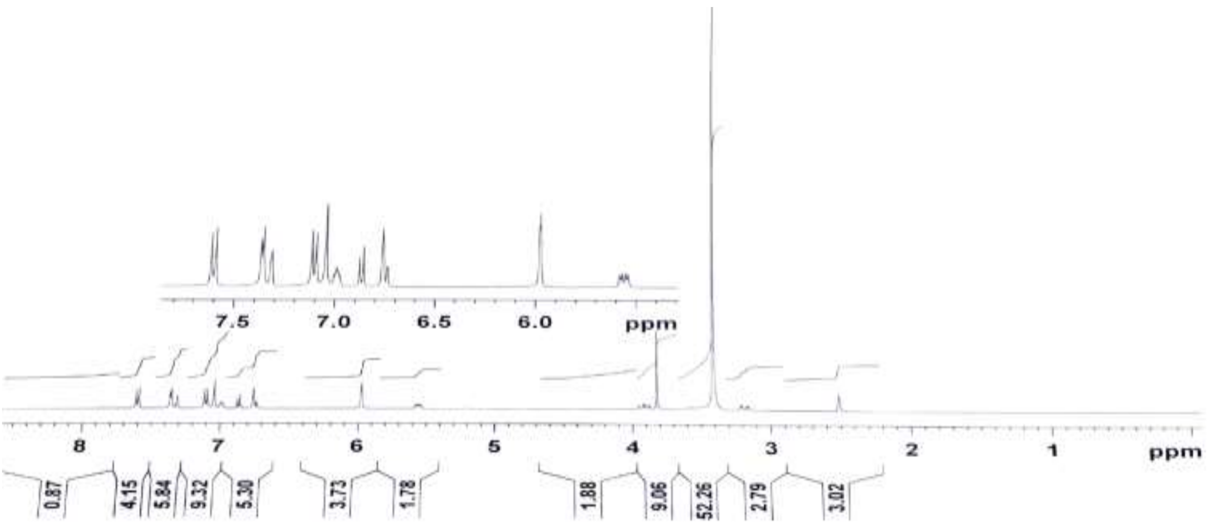

[13] C NMR of compound 4c

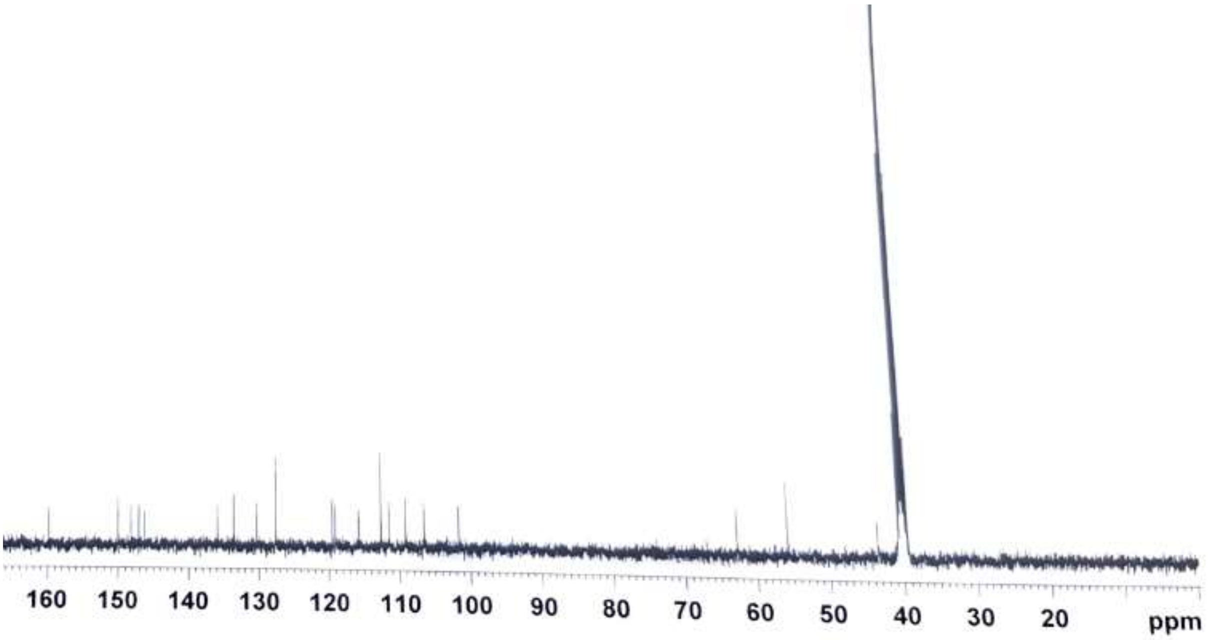

HRMS of compound 4c

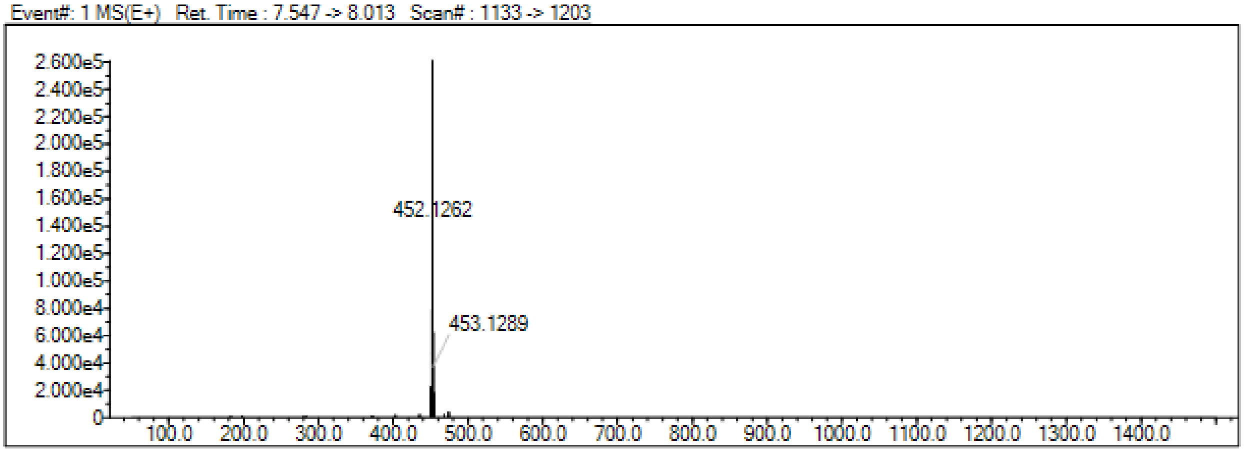

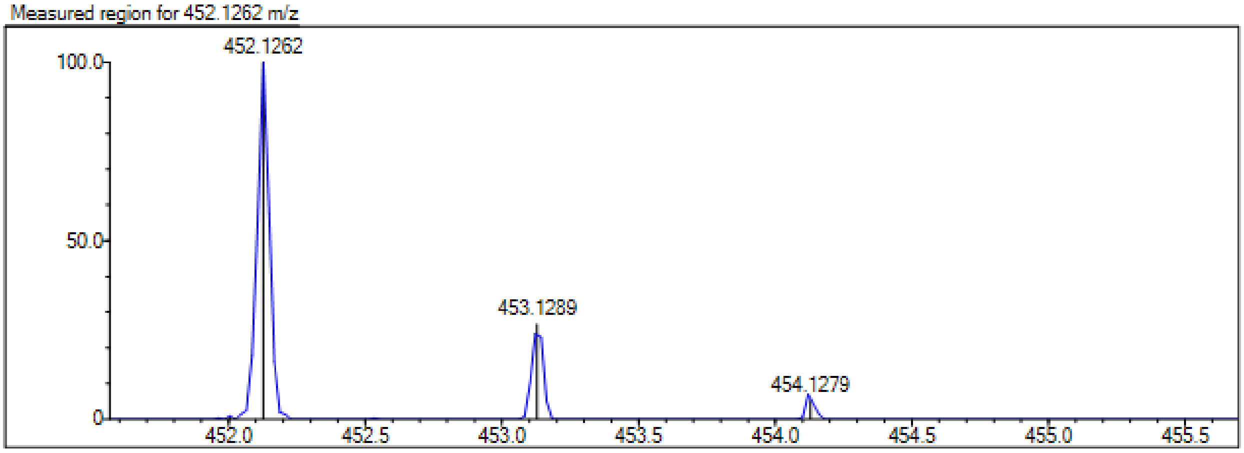

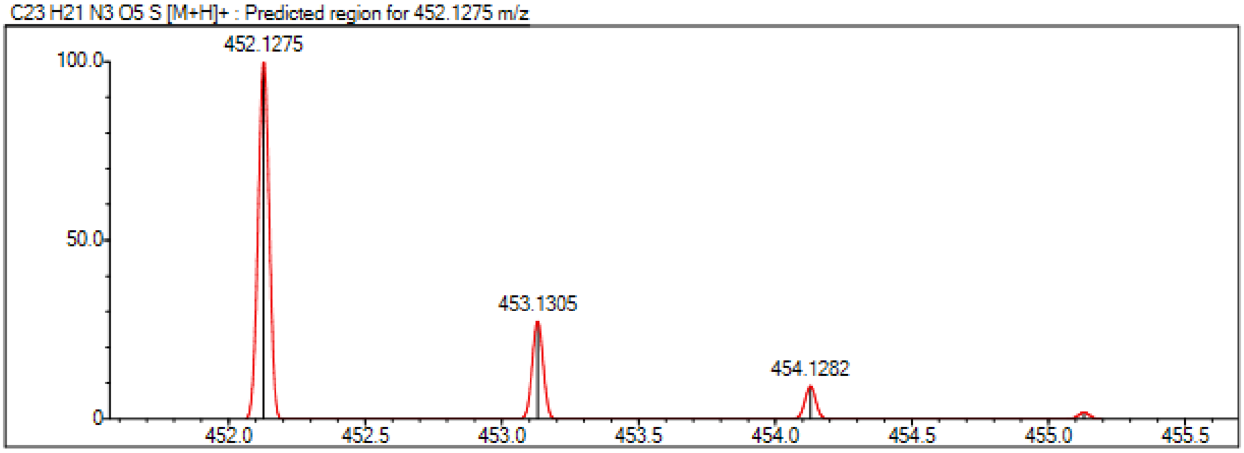

Rank	Score	Formula (M)	Ion	Meas. m/z	Pred. m/z	Df. (mDa)	Df. (ppm)	Iso	DBE
1	73.77	C23 H21 N3 O5 S	[M+H]+	452.1262	452.1275	-1.3	-2.88	77.41	15.0

Compound 4d

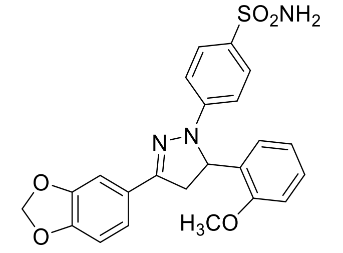

Chemical Formula: $C_{23}H_{21}N_3O_5S$
Exact Mass: 451,1202

^{1}H NMR of compound 4d

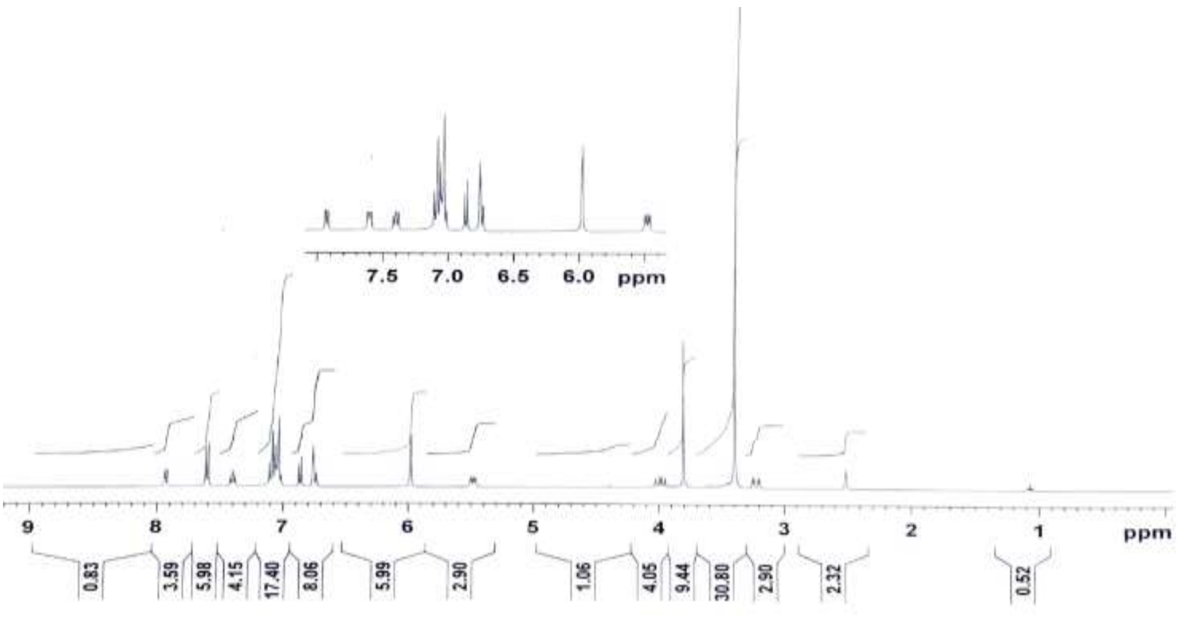

[13] C NMR of compound 4d

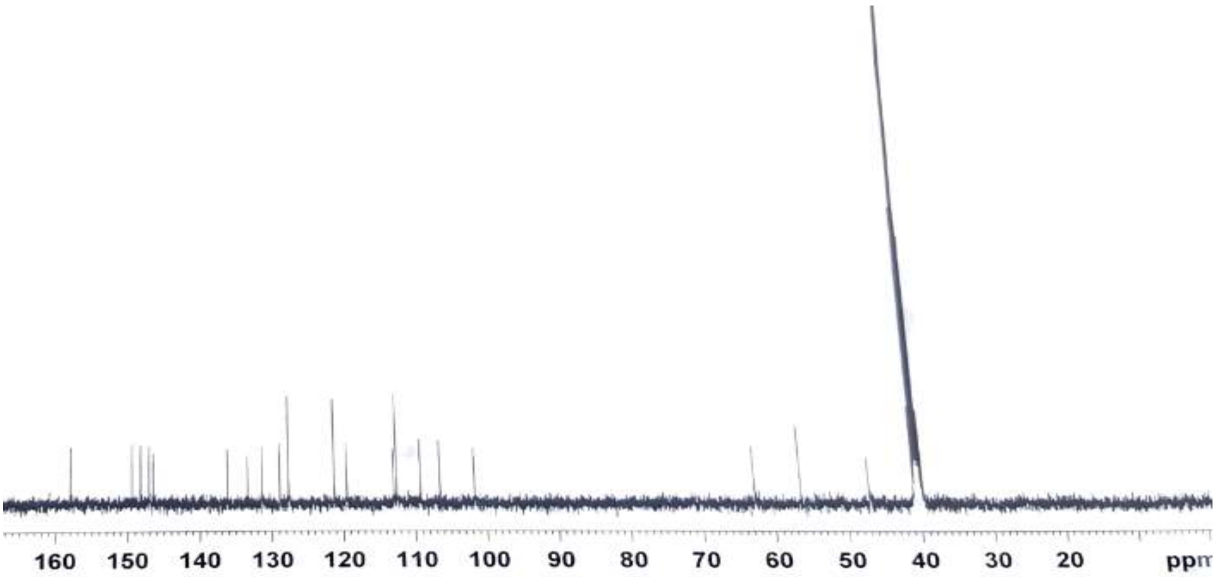

HRMS of compound 4d

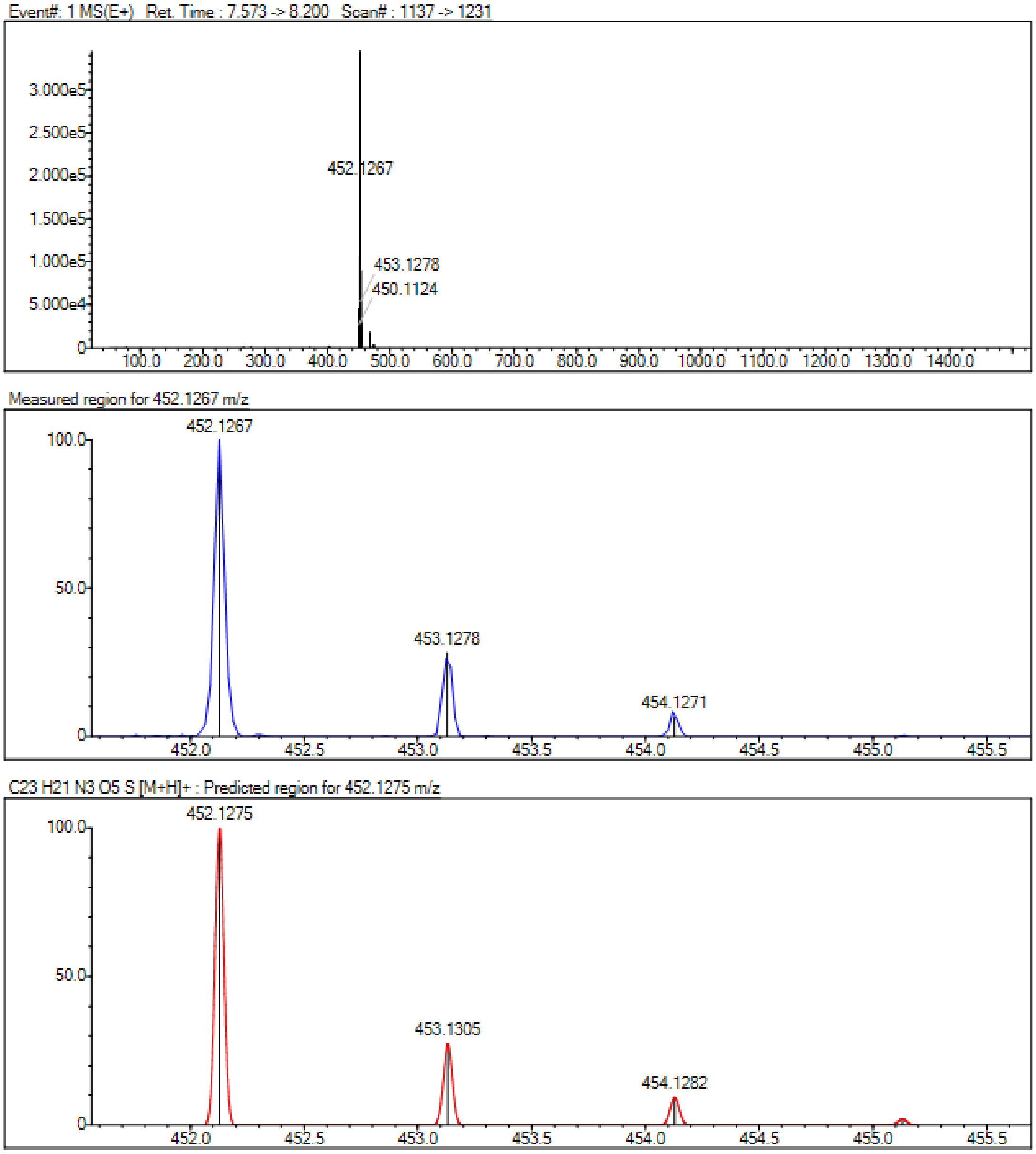

Rank	Score	Formula (M)	Ion	Meas. m/z	Pred. m/z	Df. (mDa)	Df. (ppm)	Iso	DBE
1	86.93	C23 H21 N3 O5 S	[M+H]+	452.1267	452.1275	-0.8	-1.77	88.64	15.0

Compound 4e

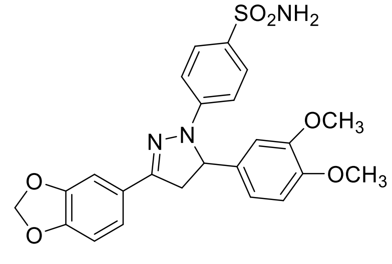

Chemical Formula: $C_{24}H_{23}N_3O_6S$
Exact Mass: 481,1308

^{1}H NMR of compound 4e

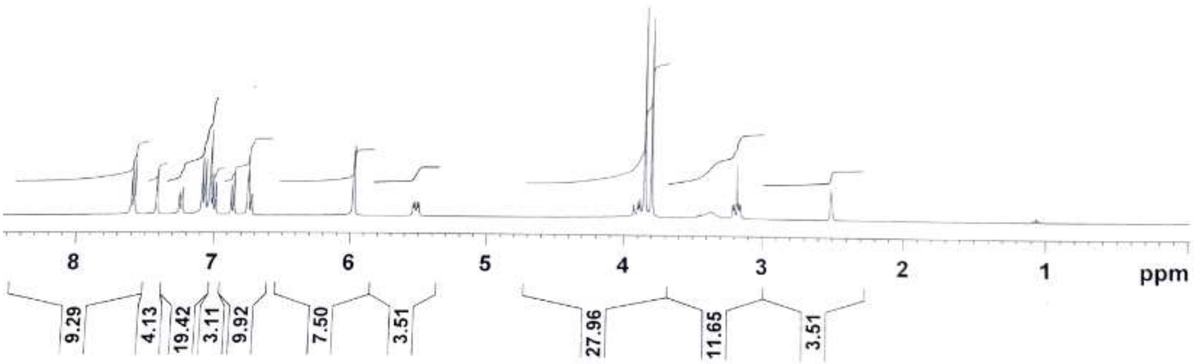

[13] C NMR of compound 4e

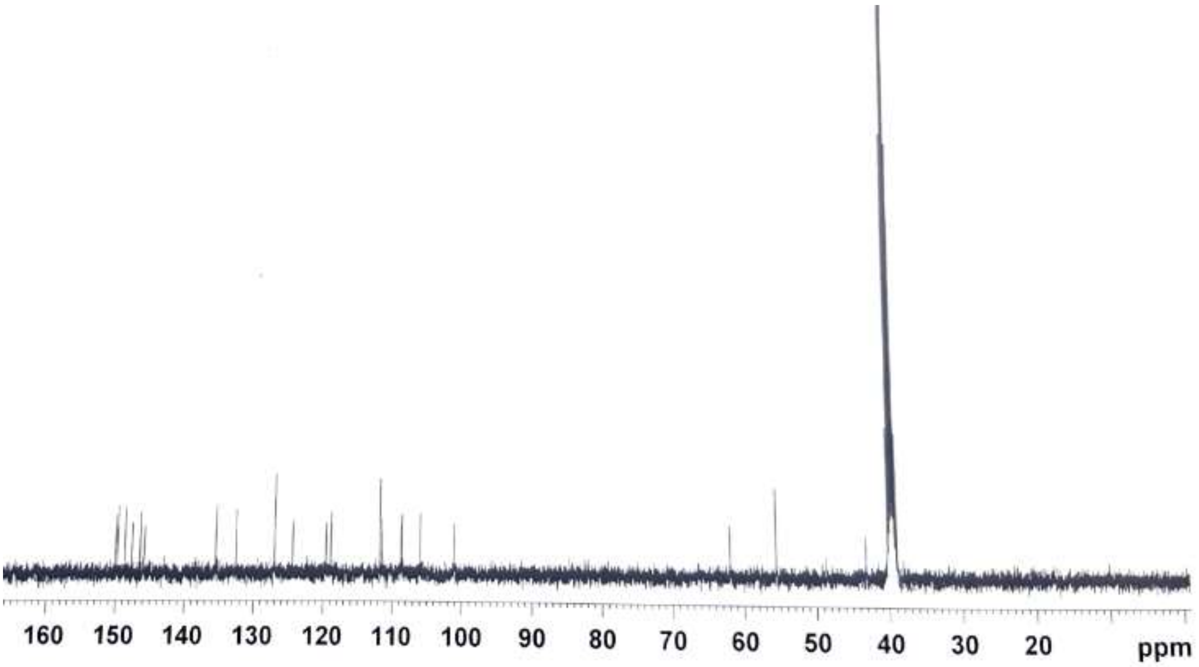

HRMS of compound 4e

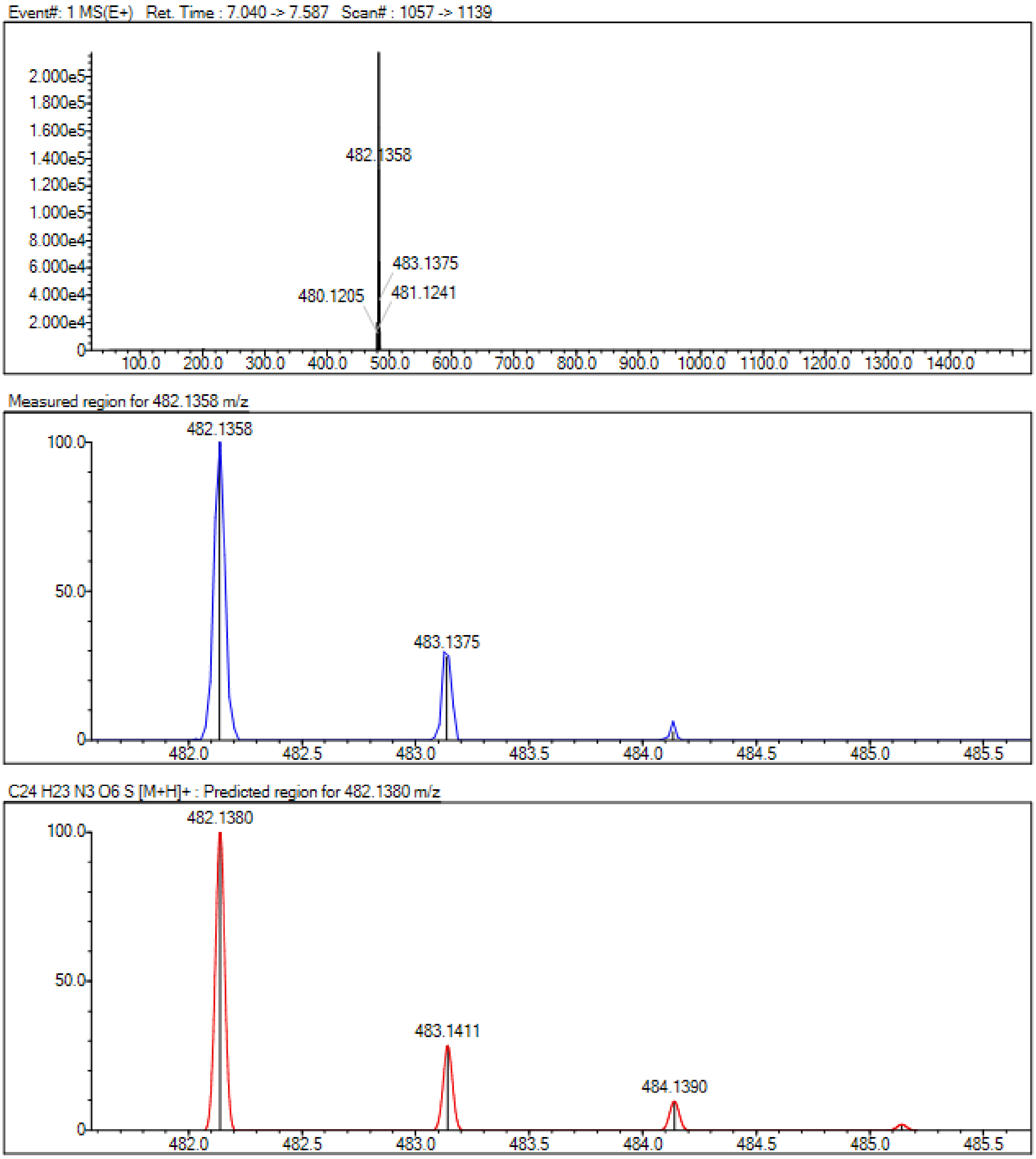

Rank	Score	Formula (M)	Ion	Meas. m/z	Pred. m/z	Df. (mDa)	Df. (ppm)	Iso	DBE
1	65.35	C24 H23 N3 O6 S	[M+H]+	482.1358	482.1380	-2.2	-4.56	71.74	15.0

Compound 4f

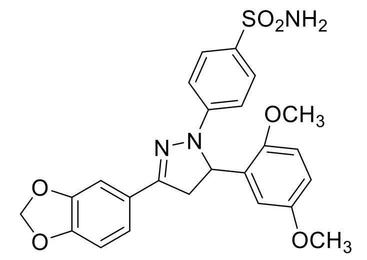

Chemical Formula: $C_{24}H_{23}N_3O_6S$
Exact Mass: 481,1308

^{1}H NMR of compound 4f

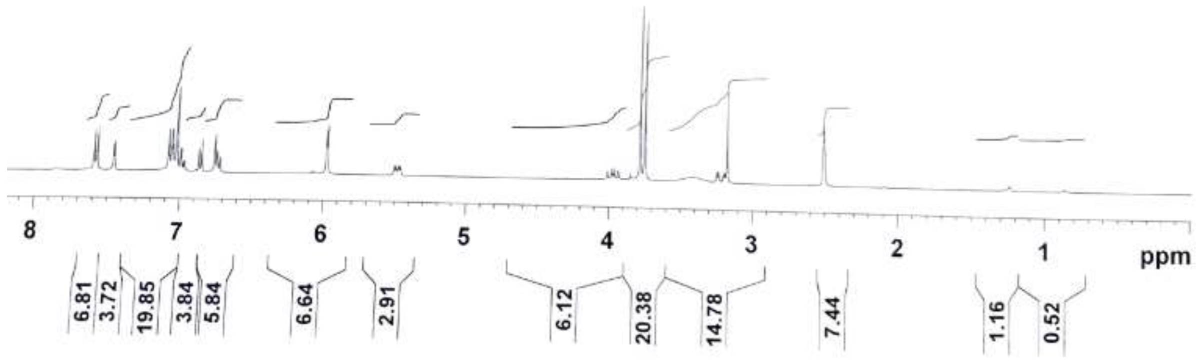

[13] C NMR of compound 4f

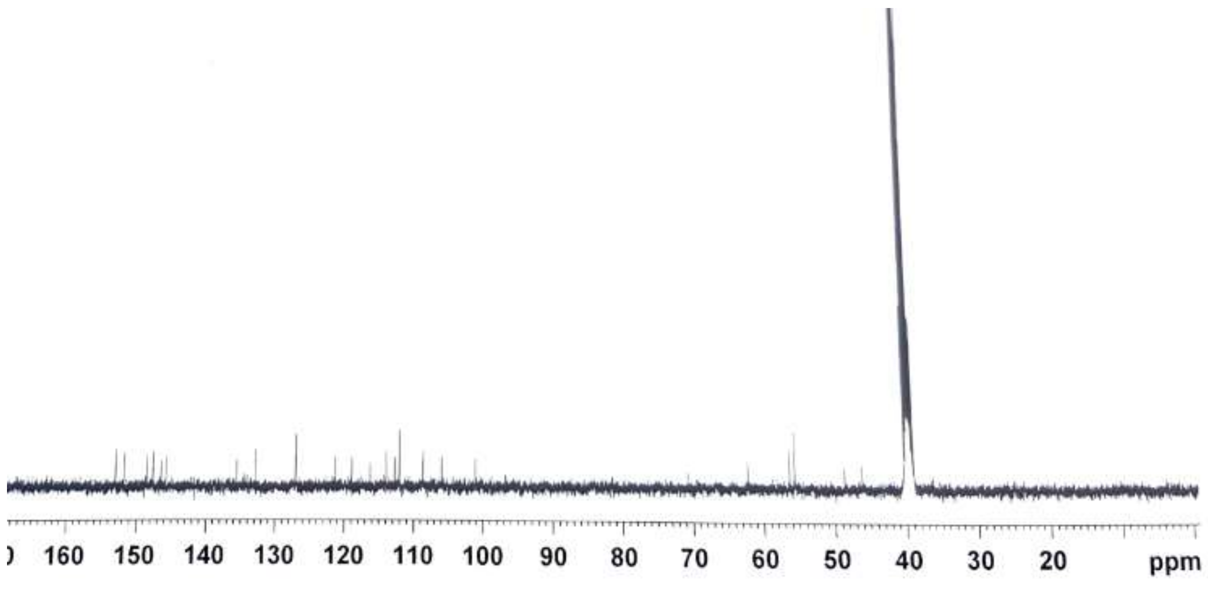

HRMS of compound 4f

Event#: 1 MS(E+) Ret. Time : 7.627 -> 8.053 Scan# : 1145 -> 1209

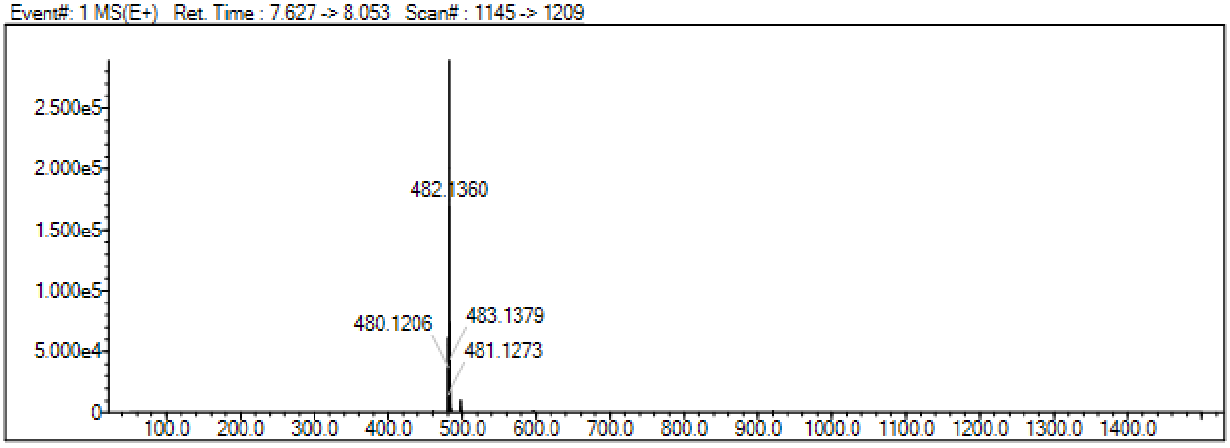

Measured region for 482.1360 m/z

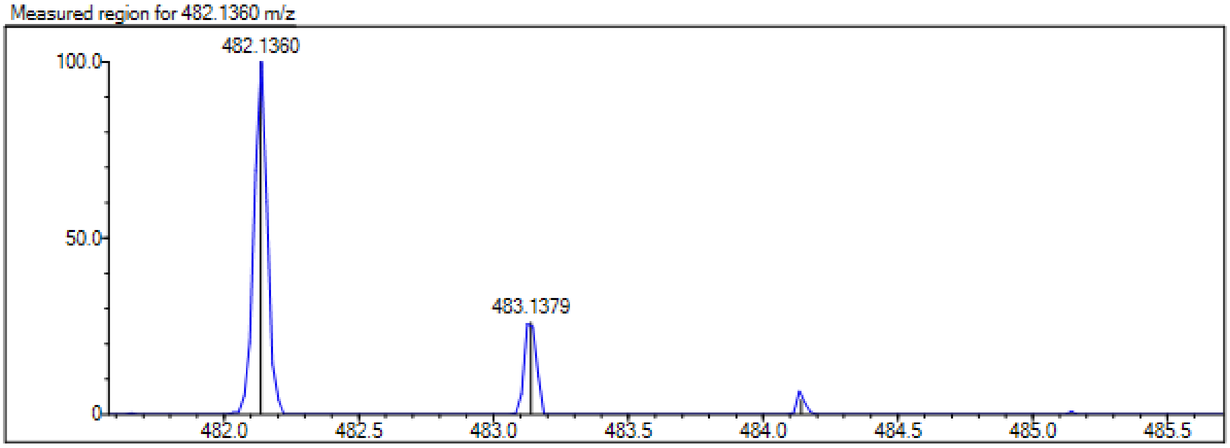

C24 H23 N3 O6 S [M+H]+ : Predicted region for 482.1380 m/z

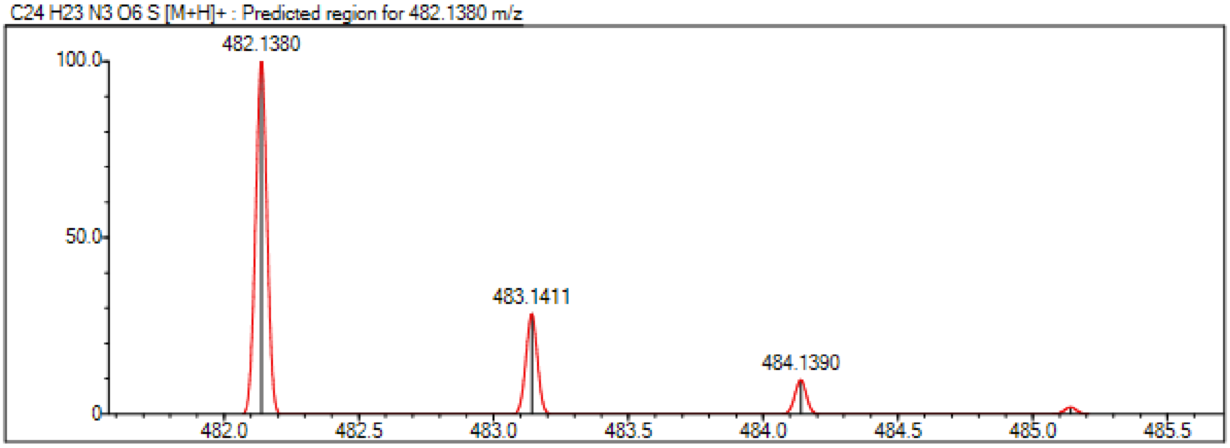

Rank	Score	Formula (M)	Ion	Meas. m/z	Pred. m/z	Df. (mDa)	Df. (ppm)	Iso	DBE
1	77.91	C24 H23 N3 O6 S	[M+H]+	482.1360	482.1380	-2.0	-4.15	84.57	15.0

Compound 4g

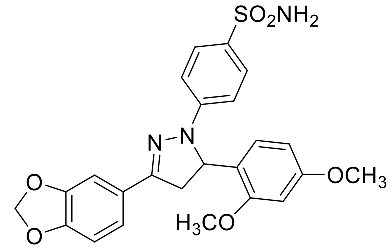

Chemical Formula: $C_{24}H_{23}N_3O_6S$
Exact Mass: 481,1308

^{1}H NMR of compound 4g

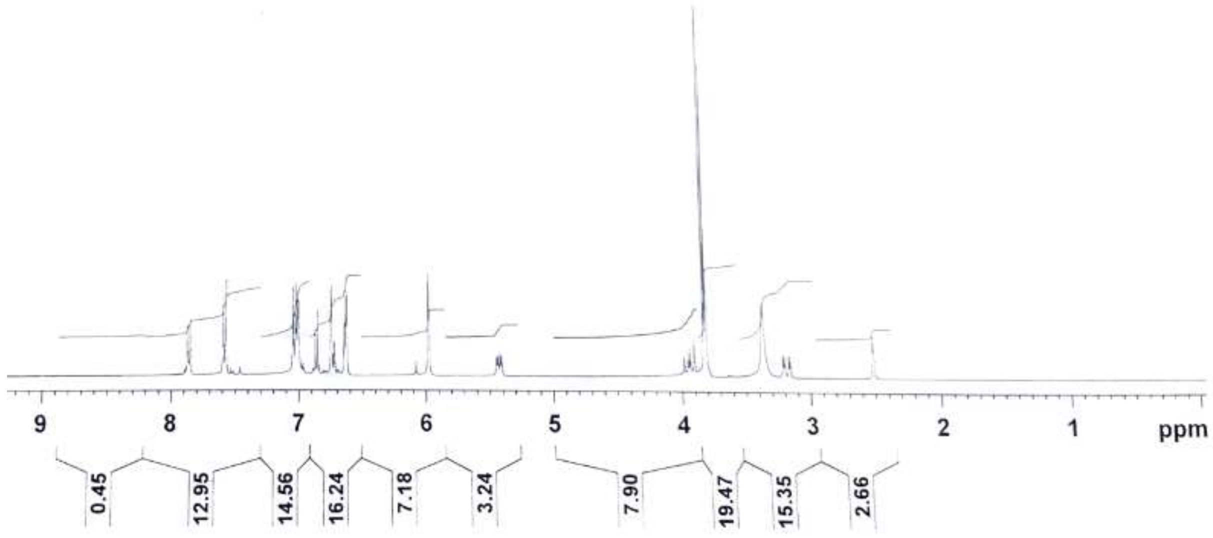

[13] C NMR of compound 4g

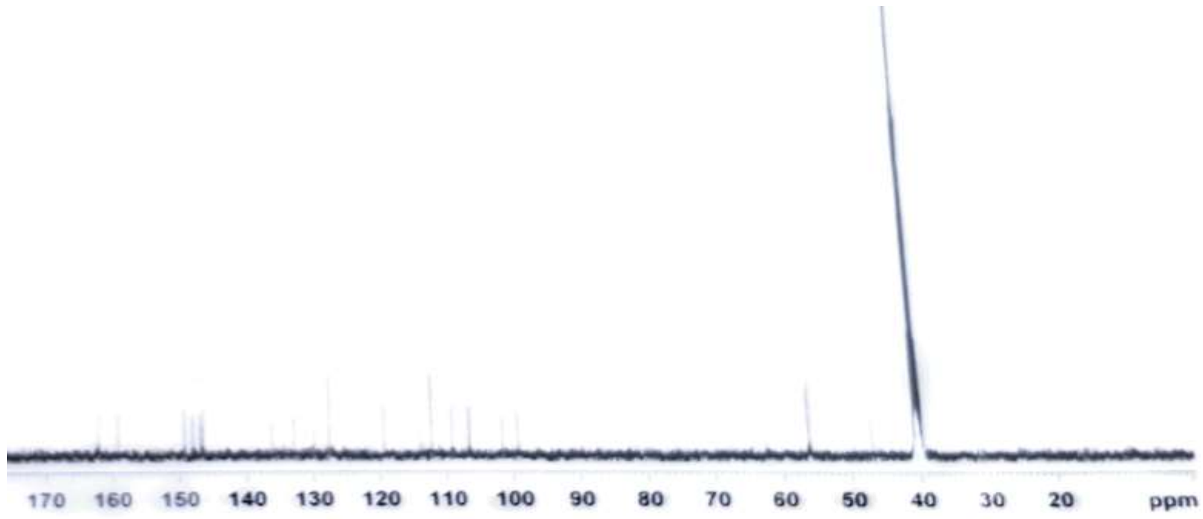

HRMS of compound 4g

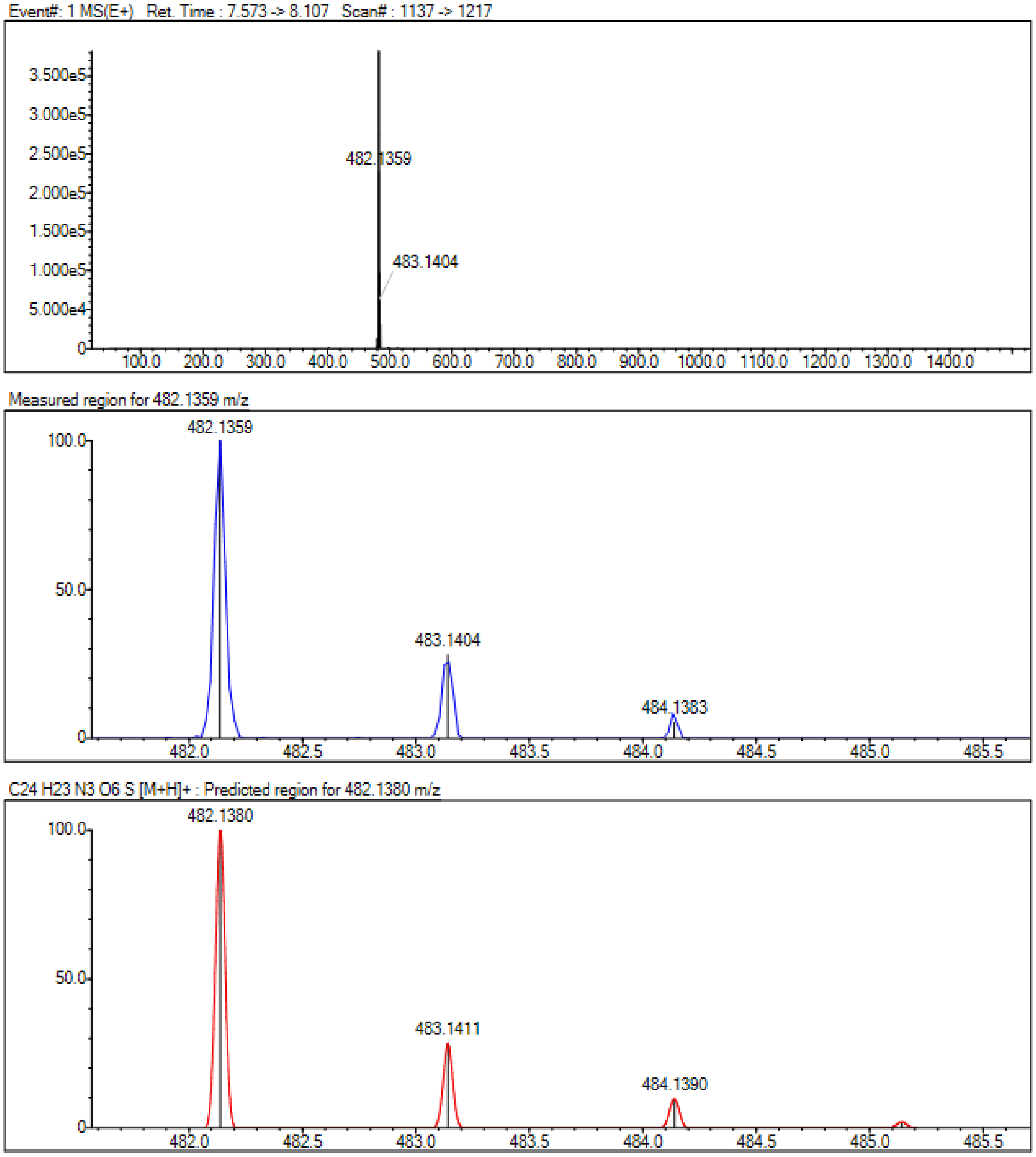

Rank	Score	Formula (M)	Ion	Meas. m/z	Pred. m/z	Df. (mDa)	Df. (ppm)	Iso	DBE
1	80.45	C24 H23 N3 O6 S	[M+H]+	482.1359	482.1380	-2.1	-4.36	87.83	15.0

Compound 4h

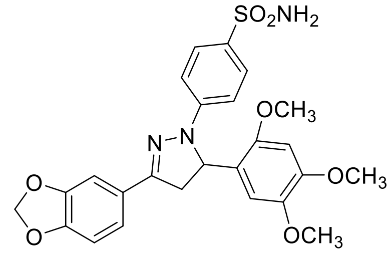

Chemical Formula: $C_{25}H_{25}N_3O_7S$
Exact Mass: 511,1413

^{1}H NMR of compound 4h

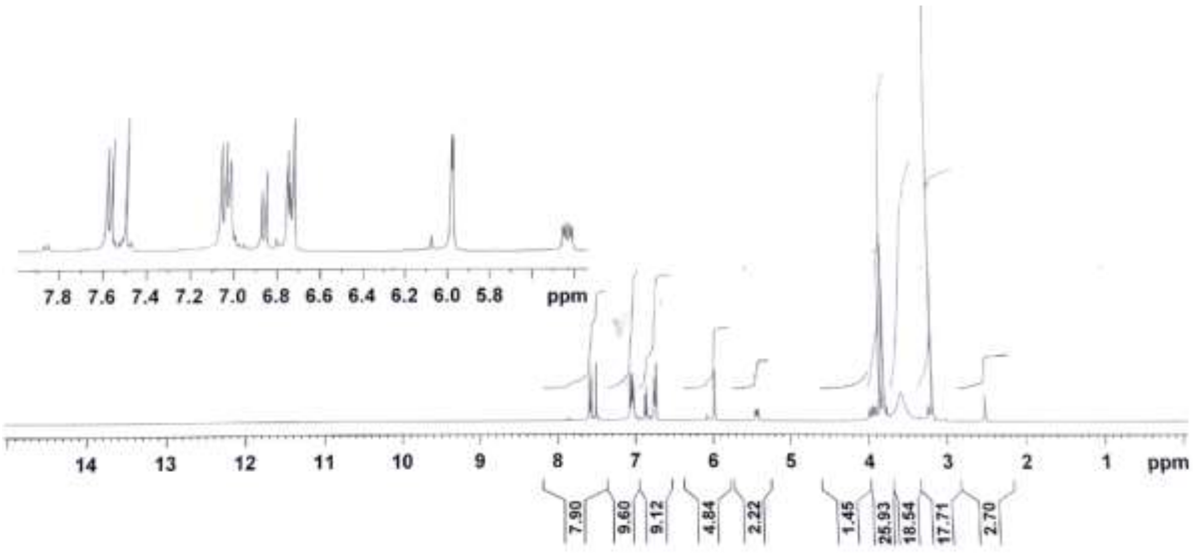

[13] C NMR of compound 4h

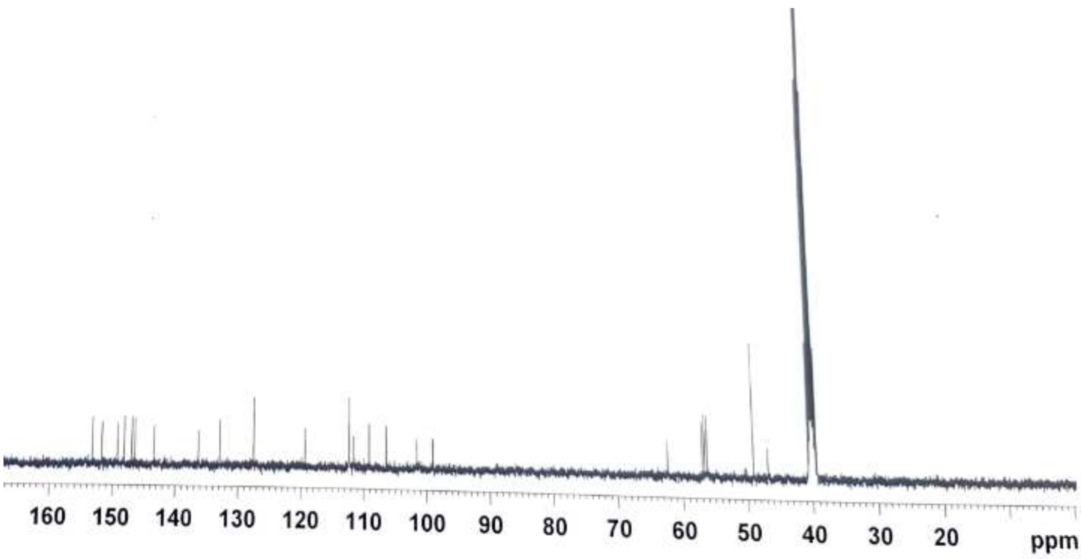

HRMS of compound 4h

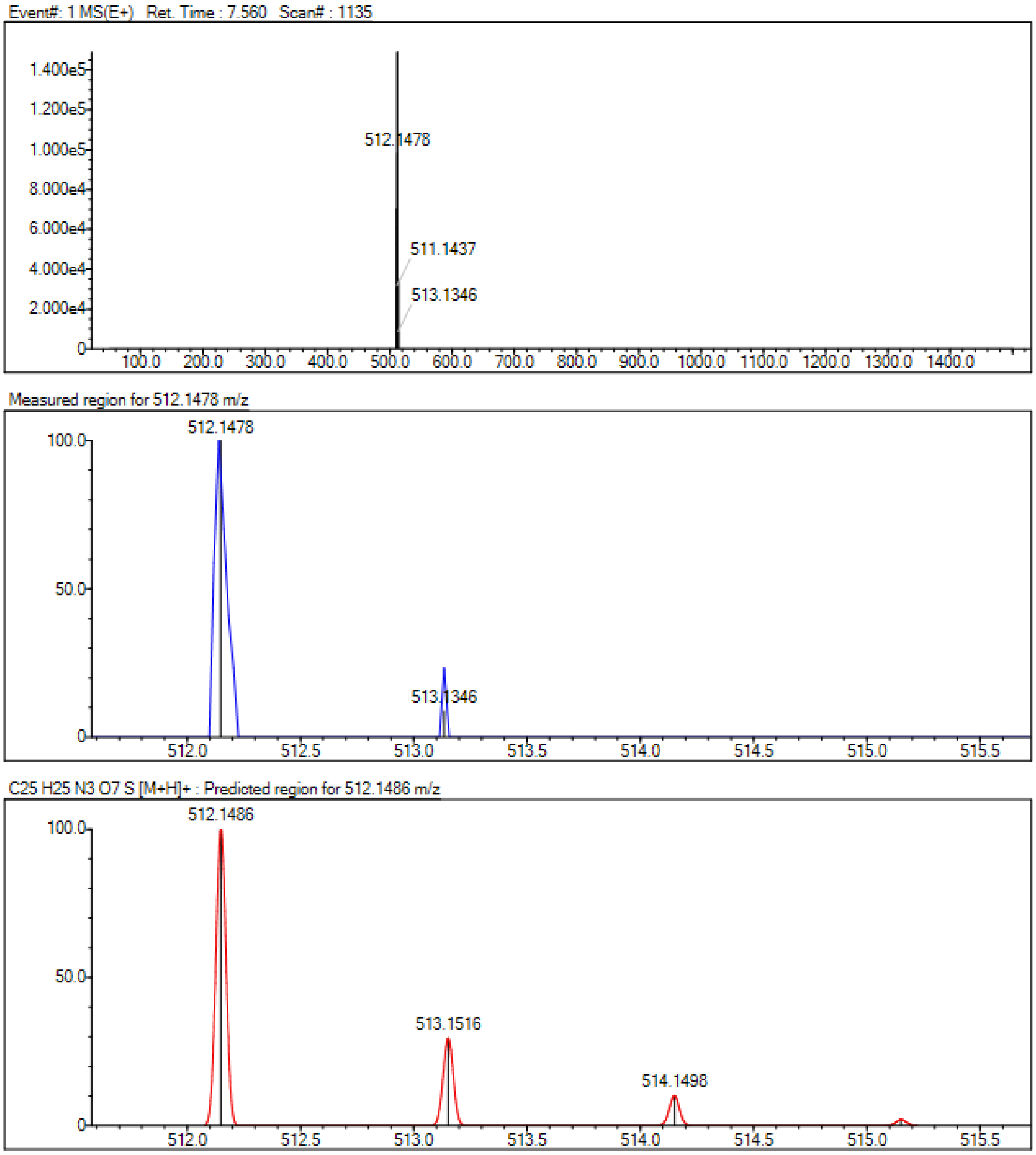

Rank	Score	Formula (M)	Ion	Meas. m/z	Pred. m/z	Df. (mDa)	Df. (ppm)	Iso	DBE
1	0.00	C25 H25 N3 O7 S	[M+H]+	512.1478	512.1486	-0.8	-1.56	0.00	15.0

Compound 4i

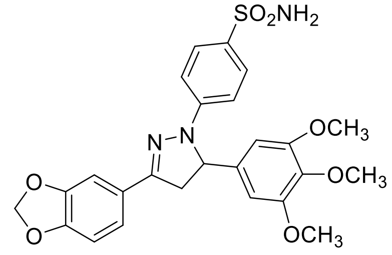

Chemical Formula: $C_{25}H_{25}N_3O_7S$
Exact Mass: 511,1413

^{1}H NMR of compound 4i

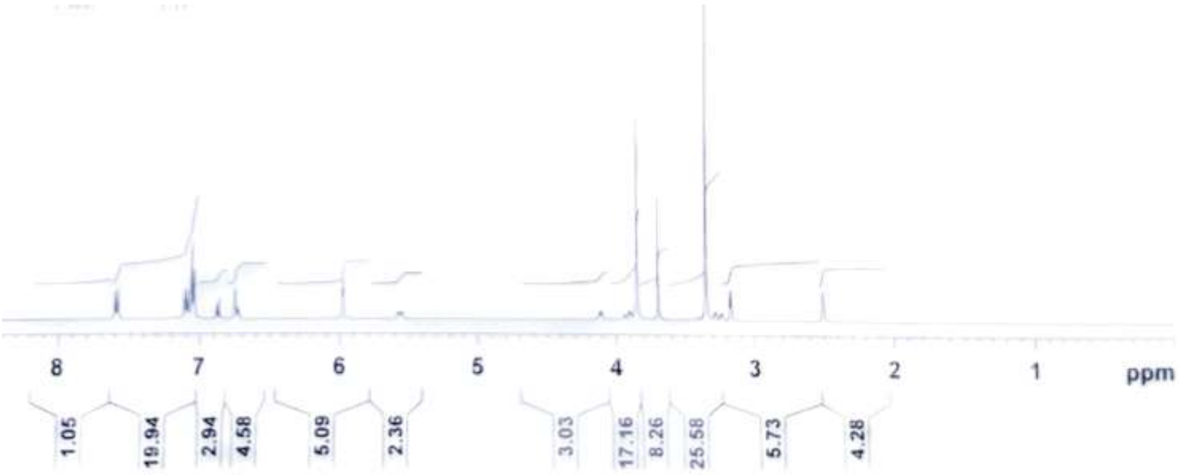

[13] C NMR of compound 4i

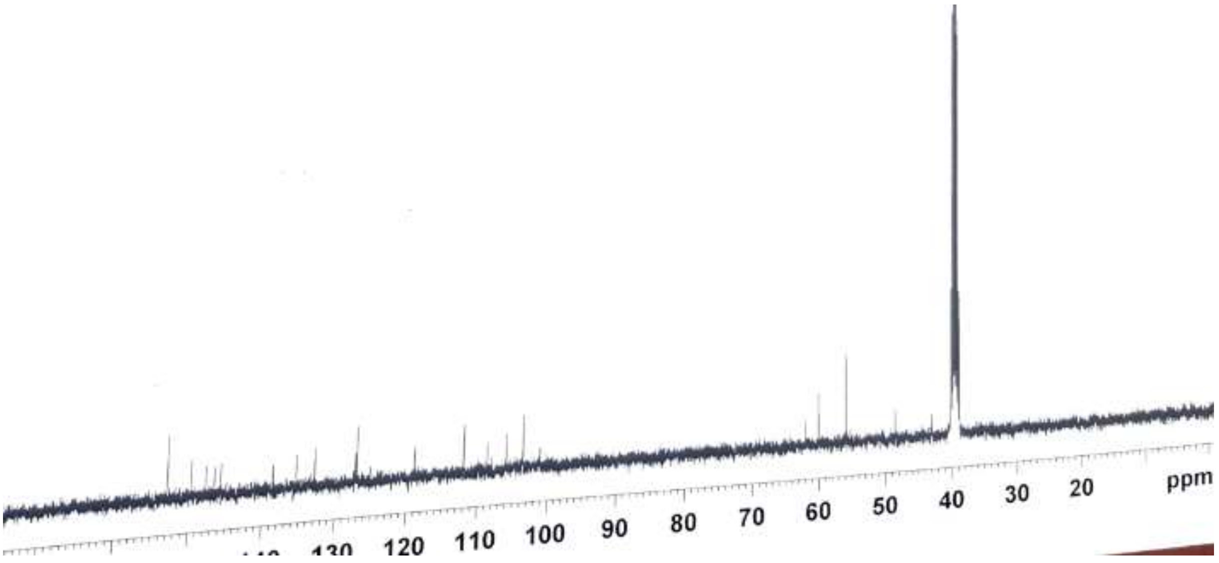

HRMS of compound 4i

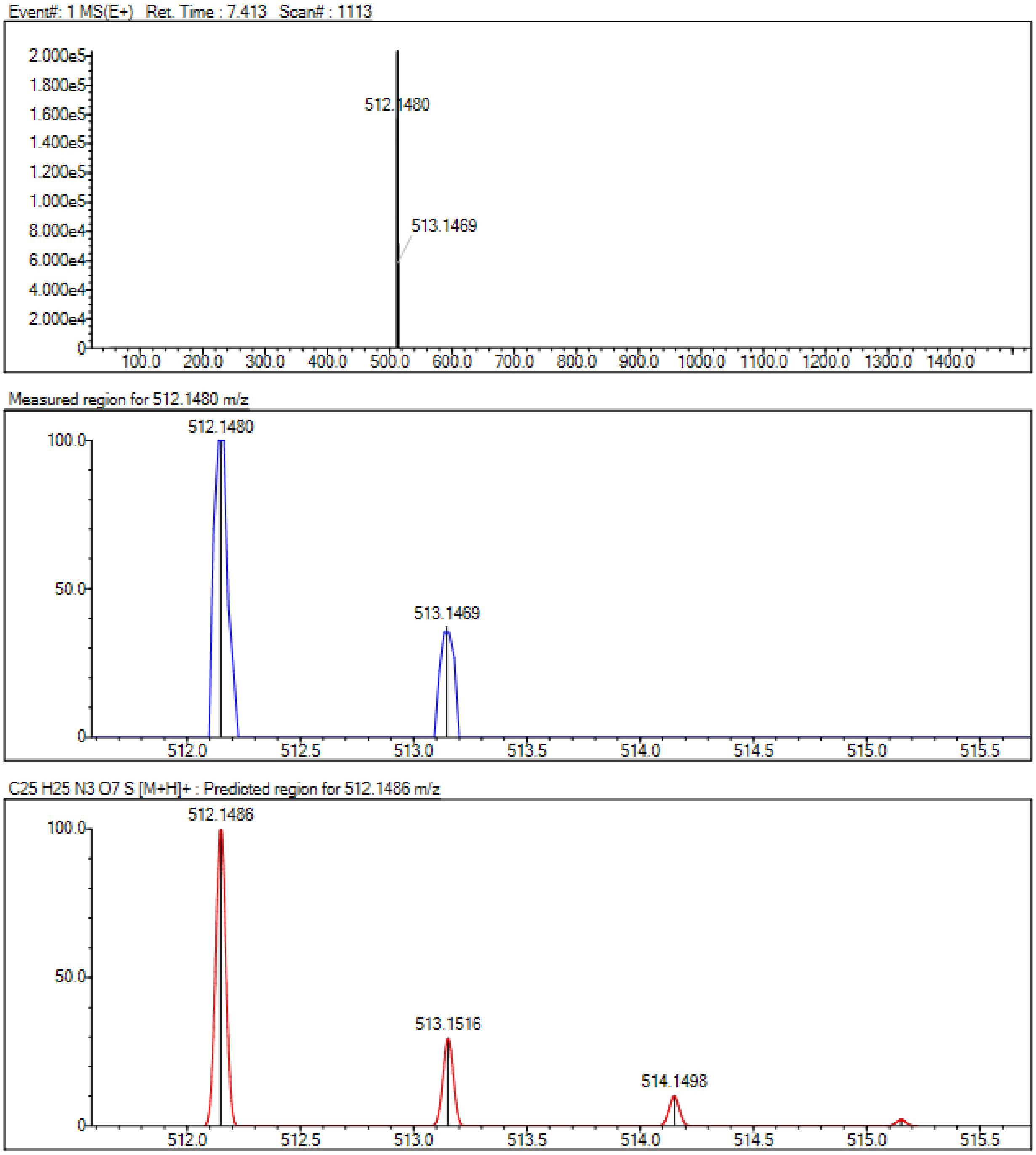

Rank	Score	Formula (M)	Ion	Meas. m/z	Pred. m/z	Df. (mDa)	Df. (ppm)	Iso	DBE
1	0.00	C25 H25 N3 O7 S	[M+H]+	512.1480	512.1486	-0.6	-1.17	0.00	15.0

Compound 4j

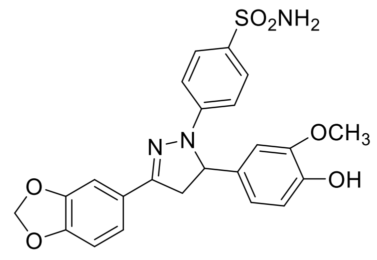

Chemical Formula: $C_{23}H_{21}N_3O_6S$
Exact Mass: 467,1151

^{1}H NMR of compound 4j

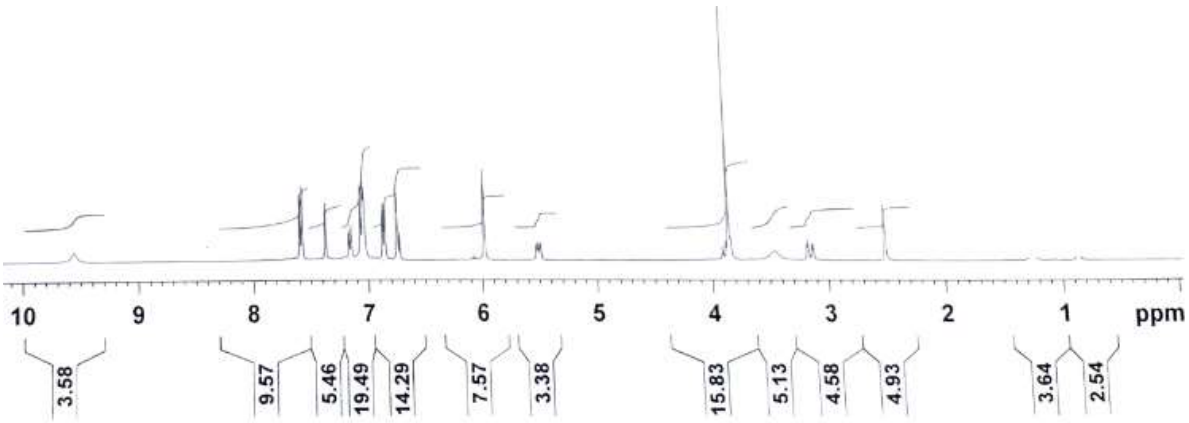

[13] C NMR of compound 4j

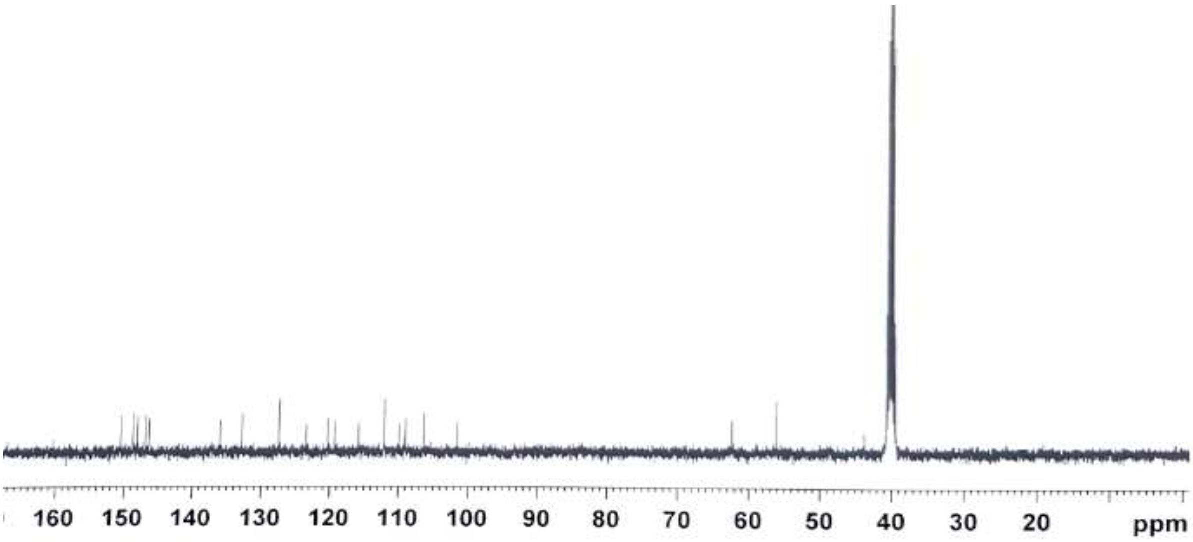

HRMS of compound 4j

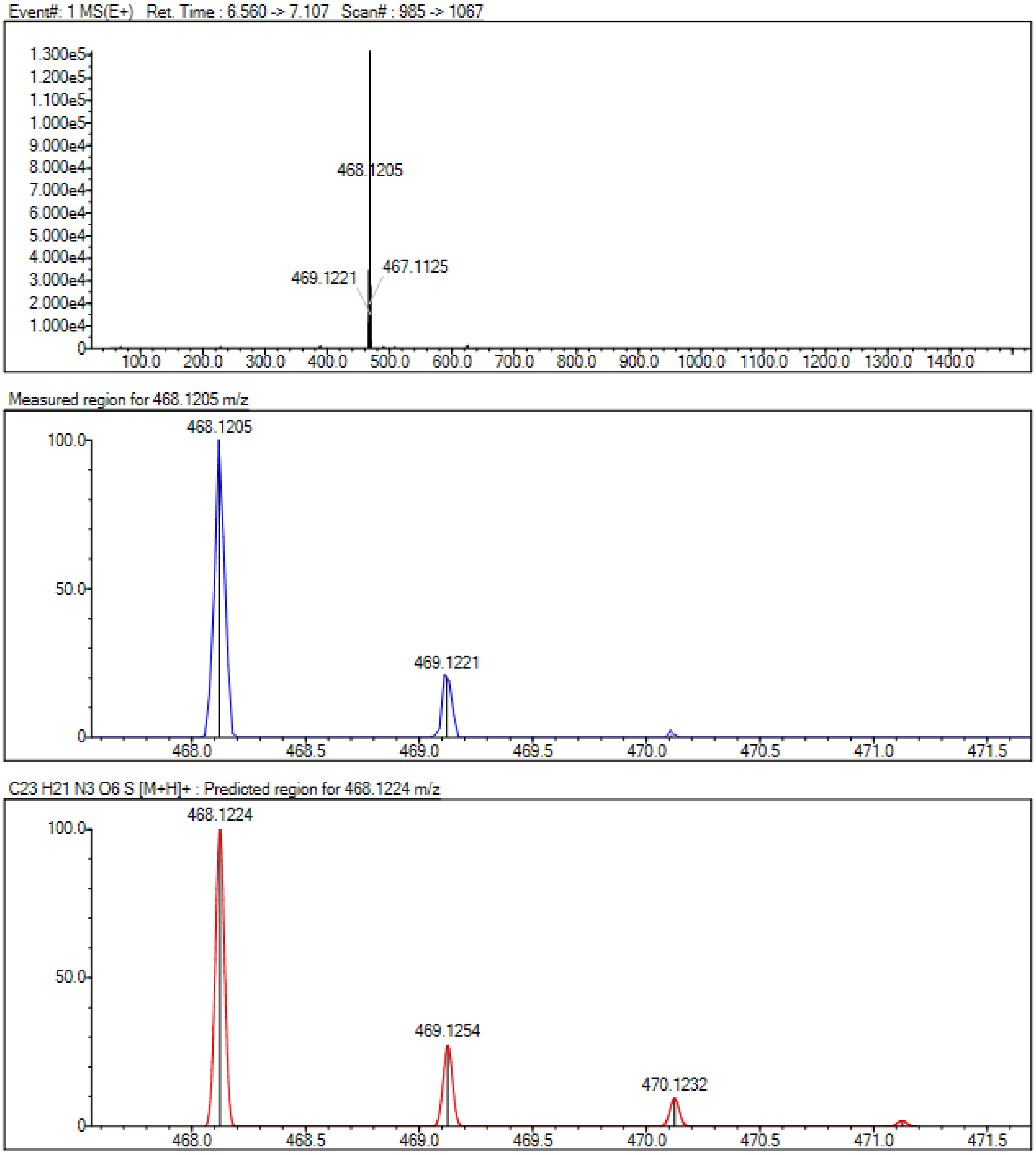

Rank	Score	Formula (M)	Ion	Meas. m/z	Pred. m/z	Df. (mDa)	Df. (ppm)	Iso	DBE
1	32.12	C23 H21 N3 O6 S	[M+H]+	468.1205	468.1224	-1.9	-4.06	34.78	15.0
